# The Intersection of Acute Kidney Injury and Non-Coding RNAs: Inflammation

**DOI:** 10.3389/fphys.2022.923239

**Published:** 2022-06-09

**Authors:** Bojun Li, Fangyou Lin, Yuqi Xia, Zehua Ye, Xinzhou Yan, Baofeng Song, Tianhui Yuan, Lei Li, Xiangjun Zhou, Weimin Yu, Fan Cheng

**Affiliations:** Department of Urology, Renmin Hospital of Wuhan University, Wuhan, China

**Keywords:** acute kidney injury, non-coding RNA, inflammation, NF-κB signaling pathway, sepsis

## Abstract

Acute renal injury (AKI) is a complex clinical syndrome, involving a series of pathophysiological processes, in which inflammation plays a key role. Identification and verification of gene signatures associated with inflammatory onset and progression are imperative for understanding the molecular mechanisms involved in AKI pathogenesis. Non-coding RNAs (ncRNAs), involved in epigenetic modifications of inflammatory responses, are associated with the aberrant expression of inflammation-related genes in AKI. However, its regulatory role in gene expression involves precise transcriptional regulation mechanisms which have not been fully elucidated in the complex and volatile inflammatory response of AKI. In this study, we systematically review current research on the intrinsic molecular mechanisms of ncRNAs that regulate the inflammatory response in AKI. We aim to provide potential research directions and strategies for developing ncRNA-targeted gene therapies as an intervention for the inflammatory damage in AKI.

## Introduction

According to the guidelines issued by the KDIGO (Kidney Disease: Improving Global Outcome) organization, acute kidney injury (AKI) is a complex clinical syndrome with systemic effects characterized by rapid impairment of glomerular filtration function (Hoste et al., 2015). The condition mainly manifests as an increase in serum creatinine concentration and a decrease in urine volume, as well as acute complications such as fluid overload, electrolyte disorders, and acid-base disorders (Ostermann et al., 2020). AKI is an independent predictor of mortality affecting 10–20% of hospitalized patients and more than 50% of critically hospitalized patients (Singbartl and Kellum, 2012; Liu K. D. et al., 2020). Simultaneously, due to the lack of effective clinical treatment strategies, the risk of patients with AKI developing chronic kidney disease and end-stage kidney disease is on the rise. Worldwide, ∼1.7 million individuals succumb to AKI every year, while its high morbidity and mortality have a significant impact on socio-economic health (See et al., 2019; Zhu et al., 2020).

The etiology of AKI is extraordinarily complex, but it is essentially related to irreversible damage to the renal parenchyma (Rossaint and Zarbock, 2016). In the clinical setting, infection or sepsis, ischemia and hypoxia, and drug nephrotoxicity are the main causes of subsequent damage to the renal organs (Bejoy et al., 2022). Renal ischemia-reperfusion injury (IRI) is the leading cause of AKI during the peri-operative period (Tang et al., 2019). The IRI-induced mismatch of nutrient and oxygen supply–and–demand in renal tissue, as well as the accumulation of toxic products and pro-inflammatory cytokines, can lead to tubular epithelial apoptosis, necrosis, and inflammation (Liu H. et al., 2019; Han and Lee, 2019). Sepsis is a clinical syndrome with high morbidity and mortality that can lead to host immune dysfunction and life-threatening organ dysfunction (Miao et al., 2020). Sepsis accounts for ∼50% of total AKI cases and up to 60% of patients with sepsis can develop AKI, significantly increasing their mortality rate (Peerapornratana et al., 2019; Pinheiro et al., 2019). In addition, the intrinsic nephrotoxicity of drugs as well as their transport and metabolism through the kidney plays a vital role in the development of acute tubular injury (Perazella, 2019). Cisplatin is currently one of the most widely studied nephrotoxic drugs, as AKI occurs in 20–40% of patients with malignant tumors that have been treated with cisplatin (Hamroun et al., 2019; Volarevic et al., 2019).

Pathologically, AKI is related to a variety of biological processes and usually described as an injury to the renal tubular epithelium and vascular system which manifests by multiple forms of programmed cell death, a delayed proliferation of renal resident cells, and an intense intrarenal inflammatory response (Mehta et al., 2007; Thomas et al., 2015). In addition to the injury-promoting mechanisms, some cellular self-protection mechanisms such as autophagy are also present in AKI (Kaushal and Shah, 2016). One of the most important features of AKI is the excessive infiltration of inflammatory cells and the massive production of inflammatory factors leading to necrosis and apoptosis of renal tubular epithelial cells (Lan et al., 2016). However, the mechanisms underlying the development and regulation of the inflammatory response in AKI are extremely complex and have not been fully elucidated.

Non-coding RNAs (ncRNAs) account for ∼90% of non-coding sequences transcribed in the human genome, and less than 2% of transcribed coding genes translate into functional proteins (Esteller, 2011). Nevertheless, between the steps of gene expression and protein translation, abnormal expression of ncRNAs influences the inflammatory response associated with AKI. Existing studies have shown that ncRNAs may regulate inflammation in AKI by inhibiting target gene translation and inducing target gene messenger RNA (mRNA) degradation (Lv et al., 2020). Therefore, elucidating the specific genetic signatures, functional roles, and internal molecular regulatory mechanisms of ncRNAs associated with the inflammatory response can facilitate the discovery of potential therapeutic targets and strategies for AKI.

### Inflammation in Acute Kidney Injury

**FIGURE 1:**
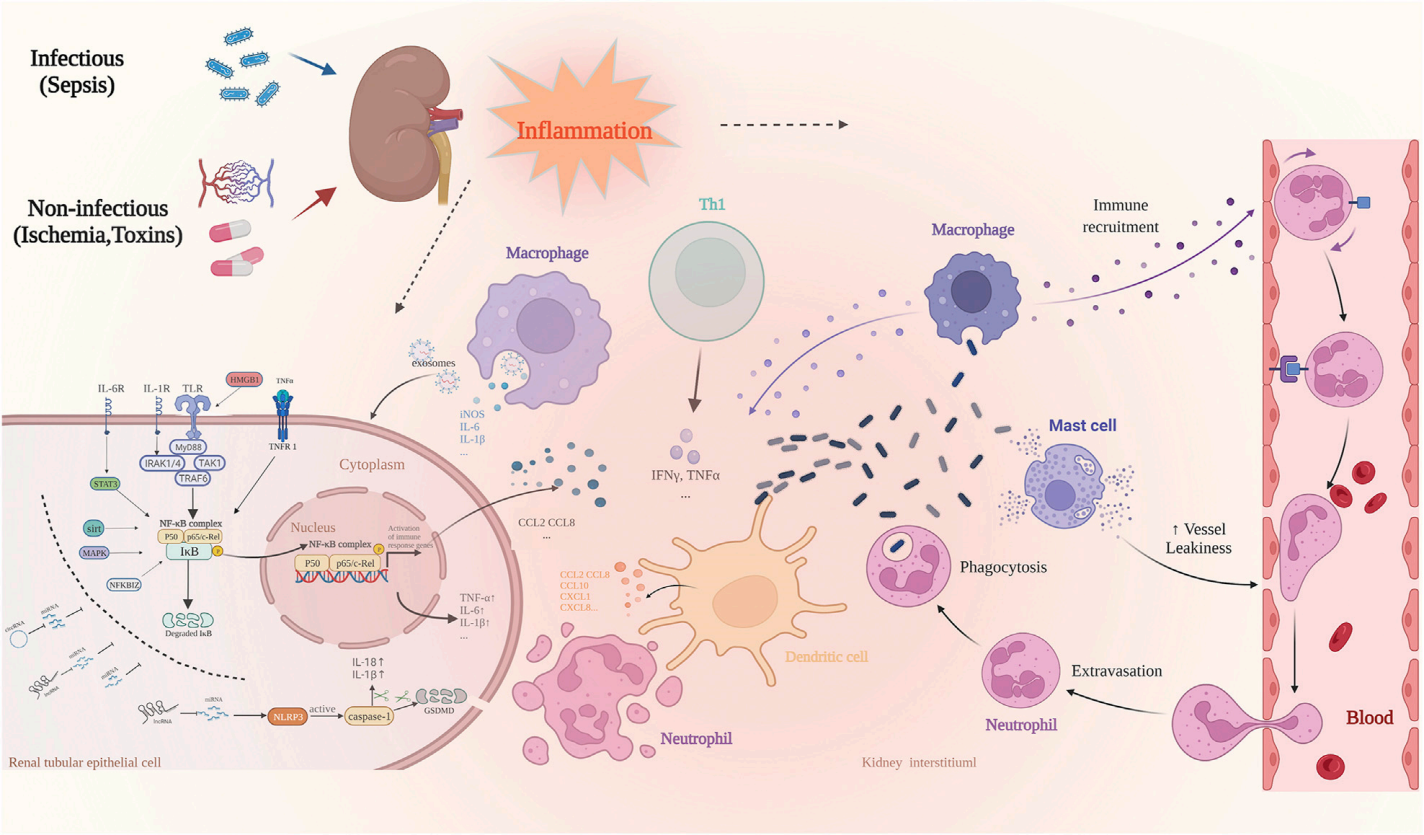
The most common causes of AKI include sepsis, ischemia-reperfusion, and nephrotoxic drug injury. It is involved in a variety of pathophysiological processes, of which inflammatory response is one of the most important pathological features. When AKI occurs, the damaged renal tubular epithelial cells increase the permeability of vascular endothelial cells by releasing pro-inflammatory cytokines, whereas activated renal tubular epithelial cells and dendritic cells secrete chemokines such as CCL10 and CXCL1, followed by a large number of immune effector cells into circulation. These include neutrophils, monocytes, and T cells, which are recruited to the site of injury. The resident and recruited immune cells simultaneously secrete pro-inflammatory mediators such as TNF-α, IFN-γ, IL-6, and IL-1β, and the induced inflammatory cascade further aggravates the damage of renal tubular epithelial cells.Many signal transduction pathways are involved in the regulation of inflammation-related genes at the transcription level. The most important is the NF-κB pathway. NcRNAs play an important role in regulating the inflammatory response of AKI, and regulate the acute inflammatory response in the kidneys *via* a variety of targets as enhancers or inhibitors.

AKI is considered an inflammatory disease with systemic effects (Kher and Kher, 2020). Inflammation is a complex biological response critical for the elimination of microbial pathogens and tissue repair after injury (Rosales, 2018). Therefore, an improved understanding of the cellular and molecular mechanisms behind the inflammatory response of AKI could unveil effective therapies for its prevention or amelioration (Wang et al., 2020b). Over the past decade, increasing research has elucidated the underlying mechanism of AKI-related inflammation (Glass et al., 2010; Van Opdenbosch and Lamkanfi, 2019). Excessive infiltration of inflammatory cells, as well as a massive production of inflammatory factors leading to necrosis and apoptosis of the renal tubular epithelial cells, are among the main pathological features of AKI (Sato et al., 2020). This excessive and uncontrolled inflammatory response leads to irreversible tissue damage (Li J. et al., 2021). During AKI, an intense inflammatory response occurs in the kidneys potentially harming other tissues and organs by releasing soluble mediators or reintroducing activated leukocytes into the circulatory system (Godin et al., 2015; Rabb et al., 2016). Thus, it is clear that the inflammatory response involved in the pathogenesis of AKI not only affects kidney function but may also lead to the development of systemic damage. Based on the cellular and molecular mechanisms associated with the pathophysiological processes of the inflammatory response, the development of new therapies for reducing the initial inflammatory kidney damage and enhancing subsequent repair and regeneration is one of the most promising avenues for the treatment of AKI (Figure 1).

### Inflammatory Cells

The complex interaction between the renal parenchyma and the circulatory system is closely related to the pathogenesis of AKI (Sato and Yanagita, 2018; Giménez-Arnau et al., 2021). The rapid infiltration of inflammatory cells from the circulatory system into the renal parenchyma is one of the main causes of renal inflammatory injury (Baek, 2019). During the inflammatory response, the injured tubular epithelial cells increase the permeability of the vascular endothelium by releasing pro-inflammatory cytokines (Zhang D. et al., 2020). This is followed by the recruitment of a large number of circulatory immune effector cells such as neutrophils, monocytes, and T cells to the injury site. The recruitment of immune cells further triggers an inflammatory cascade that aggravates the injury, creating a vicious cycle (McWilliam et al., 2021).

External stimuli can directly activate pathogen-associated molecular pattern (PAMP) recognition receptors of the tubular epithelial cells and innate immune cells residing in the kidney (Fani et al., 2018). The most common PAMP receptors include toll-like receptors (TLRs), Nod-like receptors (NLRs), and the receptor for advanced glycation end product (RAGE) (Ozkok and Edelstein, 2014; Poston and Koyner, 2019). Subsequently, these stimuli cause the production of pro-inflammatory cytokines, chemokines, and reactive oxygen species (ROS) by activating a series of signaling events (Zhao M. et al., 2021). In turn, this leads to cellular necrosis and tissue damage. In ischemic AKI, the aseptic inflammatory response associated with damage-associated molecular patterns (DAMPs) is considered one of the first pathological processes (Hepokoski et al., 2021; Liu et al., 2021). Renal tubular cell necrosis releases intracellular molecules such as high mobility group box 1 (HMGB1), histones, heat kinin, and fibronectin into the extracellular space and activates recognition receptors on either tissue-resident cells or recruited leukocytes *via* the DAMPs (Wang et al., 2020c; Gao et al., 2021). Cell necrosis then leads to a massive secretion of pro-inflammatory cytokines and chemokines, further aggravating the inflammatory cascade response.

At different stages of AKI injury, quiescent macrophages undergo phenotypic polarization influenced by the microenvironment to differentiate into two functional states with specific roles (Kormann et al., 2020). In the early stages of AKI, during the acute inflammatory response, M1 macrophages predominate, releasing pro-inflammatory mediators that cause damage to the proximal tubules in the renal cortical layer (Kusmartsev, 2021). As the disease progresses, macrophages change from pro-inflammatory M1 to anti-inflammatory M2 macrophages. M2 macrophages then release inflammatory factors related to tissue repair, such as transforming growth factor-β (TGF-β), involved in aggregating and phagocytosing apoptotic cells (He et al., 2017). It can be said that M1 macrophages are the “culprits” of tubular epithelial cell apoptosis and tubular injury, while M2 macrophages can alleviate tubular necrosis and initiate the repair process. Notably, however, macrophages can also promote renal fibrosis, which is a major driver of progression to end-stage AKI. For instance, recent studies suggest that the role of M2 macrophages in chronic kidney disease may be the opposite of that in AKI, and targeting signal transducer and activator of transcription 6 (STAT6) can inhibit the polarization of M2 macrophages, thereby protecting renal function (Jiao et al., 2021a; Jiao et al., 2021b). Elucidating the exact role and timing of M2 activation is thus imperative to stunt the progression of AKI.

### Inflammatory Cytokines

Chemokines and their receptors are crucial in the inflammatory response and can promote acute neutrophil- and monocyte–macrophage-dependent inflammatory responses, which are closely associated with the inflammatory damage in AKI (Holdsworth and Gan, 2015; Xu et al., 2015). Early into the immune response of AKI, renal parenchymal cells and dendritic cells are activated by the DAMPs(Prasada et al., 2020). This leads to the infiltration of immune cells, such as neutrophils, monocytes, and lymphocytes, to the injury site that secretes CCL and CXCL chemokines CCL10, CCL2, CCL5, CXCL1, and CXCL8 (Kurts et al., 2013). The balance between pro-inflammatory mediators such as tumor necrosis factor-α (TNF-α), interferon-γ (IFN-γ), interleukin-1β (IL-1β), IL-6, IL-17, IL-23, complement 3 (C3), C5a, C5b, and anti-inflammatory mediators such as IL-4, IL-10, TGF-β, and heme oxygenase-1 (HO-1) are important determinants mediating both early injury and later repair (Rabb et al., 2016; Chen et al., 2018; Deng B. et al., 2021). Therefore, effective inhibition of the production and secretion of pro-inflammatory factors in the early stages of AKI may be crucial to preventing its progression.

Monocytes recruited from peripheral blood can differentiate into tissue-specific macrophages (Pidwill et al., 2020), which can be classified into two polarized types according to phenotype and secreted cytokines, i.e., classically activated M1 type and selectively-activated M2 type. The M1 type macrophages secrete inducible nitric oxide synthase (iNOS), IL-1β, IL-6, IL-12, and other chemokines in response to factors such as IFN-γ and TNF-α, causing inflammatory damage to healthy tissues (Hu Q. et al., 2021). Macrophages are polarized towards the M2 phenotype by IL-4, IL-10, IL-13, and TGF-β. In contrast, the M2 macrophages exert anti-inflammatory effects, promoting wound repair and fibrous degeneration in the later stages of the inflammatory response (Venkatachalam et al., 2015).

The balance between pro- and anti-inflammatory mediators affects the tissue repair process after acute inflammatory injury, whereas an imbalanced inflammatory response can impede normal tissue repair as well as lead to abnormal remodeling and dysfunction. Ideally, the balance between pro- and anti-inflammatory mediators ensures the later repair of the renal tissue (Song et al., 2021). However, in many cases, sustained secretion of the inflammation-associated fibrotic cytokines TGF-β and IL-13 trigger epithelial-mesenchymal transition (Wang et al., 2019; Hu J. et al., 2021). In this abnormal repair process, renal tubular epithelial cells dedifferentiate into fibroblasts, and large amounts of extracellular matrix proteins are secreted, leading to renal fibrosis and the development of chronic renal insufficiency (Sato and Yanagita, 2018).

### Inflammation-Related Signaling Pathways

As a result of the potentially devastating nature of uncontrolled inflammatory responses, the expression of inflammation-associated genes is tightly regulated at several different levels (Zhao et al., 2018). Regulation at the transcriptional level is the most prominent way to influence the expression of inflammation-associated genes. The most prominent signaling pathways involved at this level include the nuclear factor kappa-B (NF-κB), mitogen-activated protein kinase (MAPK), and STAT pathways. Activation of these pathways induces nuclear translocation of transcription regulatory factors, promoting the transcription and translation of inflammation-associated genes (Linkermann et al., 2014; Wilflingseder et al., 2020; Zhao H. et al., 2021). However, the exact regulatory mechanisms, such as how these factors precisely and timely control the transcription of a single inflammatory gene in the nucleus, remain unknown.

### NF-κB Signal Transduction Pathway

NF-κB is a critical nuclear transcription factor that regulates the inflammatory response and expression of various inflammation-related genes, including inflammatory cytokines, chemokines, and adhesion factors (Lawrence, 2009; Oeckinghaus et al., 2011). NF-κB is closely associated with cell differentiation, proliferation, and survival to plays an important role in inflammatory injury, apoptosis, and tissue regeneration in AKI (Markó et al., 2016). The NF-κB family consists of five related protein members, including p50, p52, RelA (p65), RelB, and c-Rel—which are mainly regulated by IκB and IκB kinase (IΚΚ) (Andrade-Oliveira et al., 2019). The inactive NF-κB dimer is inactivated by association with IκB protein. When cells are stimulated by various factors such as lipopolysaccharide (LPS), ROS, and the cytokines TNF-α and IL-1β, IκB is phosphorylated and rapidly degraded. This allows the free NF-κB dimer to phosphorylate and translocate to the nucleus, thus promoting the transcription of inflammation-related genes (Lawrence, 2009). Taken together, the induction of NF-κB recruitment into the nucleus and subsequent transcriptional events are precisely regulated to ensure the “right” role of target genes.

Notably, TNF-α and IL-1β both form a positive feedback loop by activating the NF-κB signaling pathway. The TNF receptor (TNFR) and IL-1 receptor (IL-1R) mediate the activation of the NF-κB pathway, leading to the transcription of downstream inflammatory cytokines (Verstrepen et al., 2008). Both of these signaling pathways are essential components of the inflammatory cascade in response to AKI.

### TLR/NF-κB Signaling Pathway

The detrimental effects of AKI cause upregulation of cytokine and chemokine expression through TLR activation on the plasma membrane of renal tubular epithelial cells. In turn, activation of these cells recruits inflammatory macrophages and natural killer cells to the site of damage, further amplifying the inflammatory response and leading to apoptosis of renal tubular epithelial cells (Zhang et al., 2019). The best-characterized receptor in this process is TLR4. Its activation recruits myeloid differentiation factor 88 (MyD88), IL-1R-associated kinase (IRAK), TNFR-associated 6 (TRAF6), and TGF-beta-activated kinase 1 (TAK1) to form a functional complex for activation of the NF-κB signaling pathway (Habib, 2021; Zhang et al., 2021). Interaction between IRAK and TRAF6 activates TAK1 which triggers proteasomal degradation by phosphorylating IκB kinase, thus releasing the NF-κB dimers translocated into the nucleus to mediate the transcription and expression of inflammatory cytokines (Verstrepen et al., 2008).

### SIRT1/NF-κB Signaling Pathway

Silent information regulator transcript 1 (SIRT1; or Sirtuin1) is a histone deacetylase with various biological functions (Jiao and Gong, 2020). It has been shown that renal tubular epithelial cells overexpressing SIRT1 indirectly inhibit inflammatory cytokine expression by decreasing NF-κB activity, thus ameliorating the cisplatin-induced inflammatory response and apoptosis (Han et al., 2021). Mechanistically, SIRT1 inhibits NF-κB activation by deacetylating the Lys310 residue on the RelA/p65 subunit or by reducing the activity of the acetyltransferase P300/CBP (Yeung et al., 2004).

### Other Signaling Pathways in Inflammation

Various other signaling pathways also play a prominent role in inflammation regulation during AKI. Adenosine 5′-monophosphate (AMP)-dependent protein kinase (AMPK), the master metabolic regulator (energy sensor) in eukaryotic cells (Carling, 2017), has been shown to play an anti-inflammatory role *via* inhibition of NF-κB activity in LPS-induced renal tubular epithelial cells (Yeung et al., 2004). MAPK, also known as p38, is a crucial integrator of multiple signaling pathways that induce IκB phosphorylation and thus degradation, which in turn activates NF-κB to participate in the inflammatory process during AKI (Wang H. et al., 2021).

Inflammatory vesicles are the primary signaling receivers of the classical pathway of cell pyroptosis and are composed of NOD-like receptor protein 3 (NLRP3), apoptosis-associated speck-like protein containing a CARD (ASC), and caspase-1 precursor (Kim et al., 2019). The two main functions of activated caspase-1 are to shear gasdermin D (GSDMD) for cleaving it into a peptide containing the active N-terminal domain to induce cell perforation and death, and to cleave the precursors of IL-1β and IL-18 to induce their activation (Xiao et al., 2020; Deng J. et al., 2021). Thus, a close relationship exists between cell pyroptosis and the inflammatory response.

### Non-Coding RNA Characteristics and Mechanisms of Action

**FIGURE 2:**
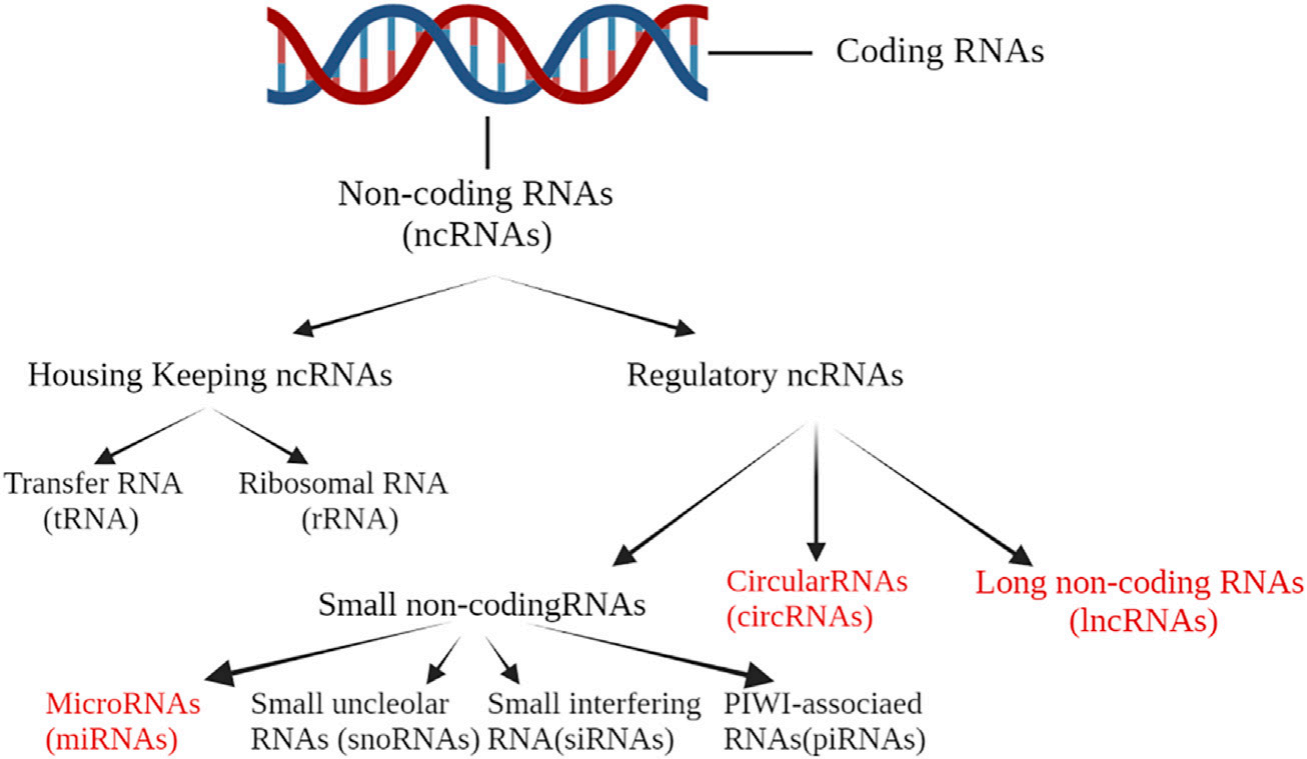
Less than 2% of the human genome is transcribed into mRNA with protein-encoding functions. For the rest of the human genome, more than 80% is transcribed into RNA without protein-encoding potential. Such functional genomic transcripts are called ncRNA. NcRNAs mainly include microRNA (miRNA), long non-coding RNA (lncRNA), circular RNA (circRNA), small nucleolar RNA (snoRNA), PIWI-associated RNAs (piRNAs) and transfer RNA (tRNA).

Less than 2% of the human genome is transcribed into mRNA with protein-encoding functions (Lin F. et al., 2022). For the rest of the human genome, more than 80% is transcribed into RNA without protein-encoding potential (Li T. et al., 2021). Such functional genomic transcripts are called ncRNAs. NcRNAs mainly include microRNA (miRNA), long non-coding RNA (lncRNA), circular RNA (circRNA), small nucleolar RNA (snoRNA), P-element-inducedwimpy testis (PIWI)-associated RNAs (piRNAs) and transfer RNA (tRNA) (Liu Z. et al., 2019). The classification of ncRNAs is summarized in Figure 2. The different ncRNAs act through specific mechanisms: miRNAs exert effects mainly by forming an RNA-induced silencing complex (RISC) to inhibit the translation of target mRNA and reduce its stability; lncRNAs participate in RNA transcription and post-transcriptional regulation through a variety of different mechanisms and interactions; and circRNAs act as either a sponge for RNA or a scaffold for transcription factors to regulate gene expression (Cech and Steitz, 2014; Ren et al., 2019).

### MiRNAs

Most miRNAs are generated *via* the classical pathway. In this pathway, primary miRNAs (pri-miRNAs) transcribed from DNA sequences are cleaved into precursor miRNAs (pre-miRNAs) at their stem-loop structure by the RNase III enzyme, drosha (Lu and Rothenberg, 2018). This cleavage is followed by the translocation of pre-miRNAs from the nucleus to the cytoplasm, where they are further cleaved by the RNase III enzyme, dicer, to produce single-stranded mature miRNAs (Saliminejad et al., 2019). Mature miRNAs are endogenous non-coding single-stranded small RNA molecules (20–25 nucleotides long). MiRNAs are named based on their directionality, with the 5p strand coming from the 5′ end of the pre-miRNA hairpin and the 3p strand from the 3′ end (Jiang and Zhu, 2020). MiRNAs target the 3′-untranslated region (3′-UTR) of mRNAs by forming the RISC 3′-UTR, repressing its expression, and participating in post-transcriptional gene regulation (Kim et al., 2012).

### LncRNAs

LncRNAs are ncRNAs (>200 nucleotides) that lack the complete open reading frame, thus lacking protein-coding potential (Lodde et al., 2020). They play an important role in gene transcription and post-transcriptional regulation. LncRNAs also regulate a wide range of biological processes such as cell differentiation, apoptosis, and inflammatory responses (Zhu et al., 2013). LncRNAs have been linked to the occurrence and development of many human diseases, and are regarded as potential new biomarkers to diagnose and predict the prognosis of diseases (Qian et al., 2019). Similar to protein-encoding mRNAs, transcription and modification of lncRNAs are usually performed by RNA polymerase II in the nucleus—hence, the presence of the 5′ cap structure and 3′ poly-A tail in their secondary structure (Yang M. et al., 2022). LncRNAs exert their biological function mainly through base pairing in the primary structure, but the functional regions such as the stem-loop structural domain in the secondary structure are key to expanding their function. Currently, the recognition of lncRNA homologs among different species remains challenging compared with the recognition of protein-encoded transcripts (Diederichs, 2014; Chen, 2016; Schmitz et al., 2016; Kopp and Mendell, 2018). In addition, expression patterns of lncRNAs show a high degree of tissue and cell specificity compared to the gene that encodes the protein (Zhou et al., 2021b).

LncRNAs play a central role in epigenetic processes and chromatin regulation. LncRNAs can interact with DNA, other RNAs, and proteins through nucleotide base pairing or a domain generated by RNA folding, which endows lncRNAs with extensive ability to regulate gene transcription and perform biological functions (Ali and Grote, 2020). Specifically, lncRNAs mainly interact with the corresponding effector proteins through the following five mechanisms to regulate gene expression: 1) lncRNAs are specifically expressed in certain cells and tissues, where they are signaled in response to unique stimuli to serve as an indicator of transcriptional activity; 2) lncRNAs can act as competitive binding sites for open chromatin, leading to transcription factor substitution or miRNA segregation, thereby derepressing target mRNAs; 3) lncRNAs guide the ribosome-protein complex to a sequence-specific binding site by binding to a target effector protein, thus mediating the transcription of specific genes; 4) lncRNAs have a “scaffold” function, aggregating multiple subunits of effector proteins or complexes through specific domains to coordinate their activities and mediate transcriptional activation or inhibition, and 5) lncRNAs can affect mRNA stability and modify chromatin structure in a similar mechanism as for miRNAs (Ferrè et al., 2016; Qian et al., 2019; Zhou et al., 2020; Bridges et al., 2021). The above mechanisms are not mutually exclusive; hence, many lncRNAs can function through multiple mechanisms simultaneously.

### CircRNAs

CircRNAs are a type of endogenous ncRNA that possess the structural characteristics of a covalently closed continuous loop (Patop et al., 2019). This loop is formed by the cis-element formed by the reverse complementary pairing sequence of the intron or by the regulation of the trans-factor consisting of RNA-binding protein (RBP) during splicing (Chen X.-T. et al., 2021). Unlike linear RNA with a 5′ cap and a 3′ ploy-A tail, the circRNA molecule has a closed-loop structure, not affected by RNA exonuclease (Chen et al., 2019). Therefore, circRNA expression is more stable and its structure not easily degraded, resulting in a highly stable cyclized sequence in intra- and extracellular environments (Yu et al., 2021). CircRNAs are widely expressed in eukaryotic tissues and organs, regulating various physiological and pathological processes in the human body (Chen L. et al., 2021). Furthermore, as a type of miRNA and lncRNA, circRNAs can also be delivered by exosomes and are readily detectable in circulation and urine, making circRNAs a potential biomarker for many diseases (Yu et al., 2021).

The function of most circRNAs remains largely elusive, but its most apparent mechanism of action is acting as a “sponge” of miRNAs to regulate gene transcription and expression (Zang et al., 2020). CircRNA molecules are enriched with miRNA binding sites, which can derepress miRNAs in the cytoplasm by competitive binding to target mRNAs(Jin et al., 2020). However, at the transcript level, the circRNAs in the nucleus mainly function through interaction with the parental genes (Kristensen et al., 2019).

### Non-coding RNAs and Inflammation in Acute Kidney Injury

**FIGURE 3:**
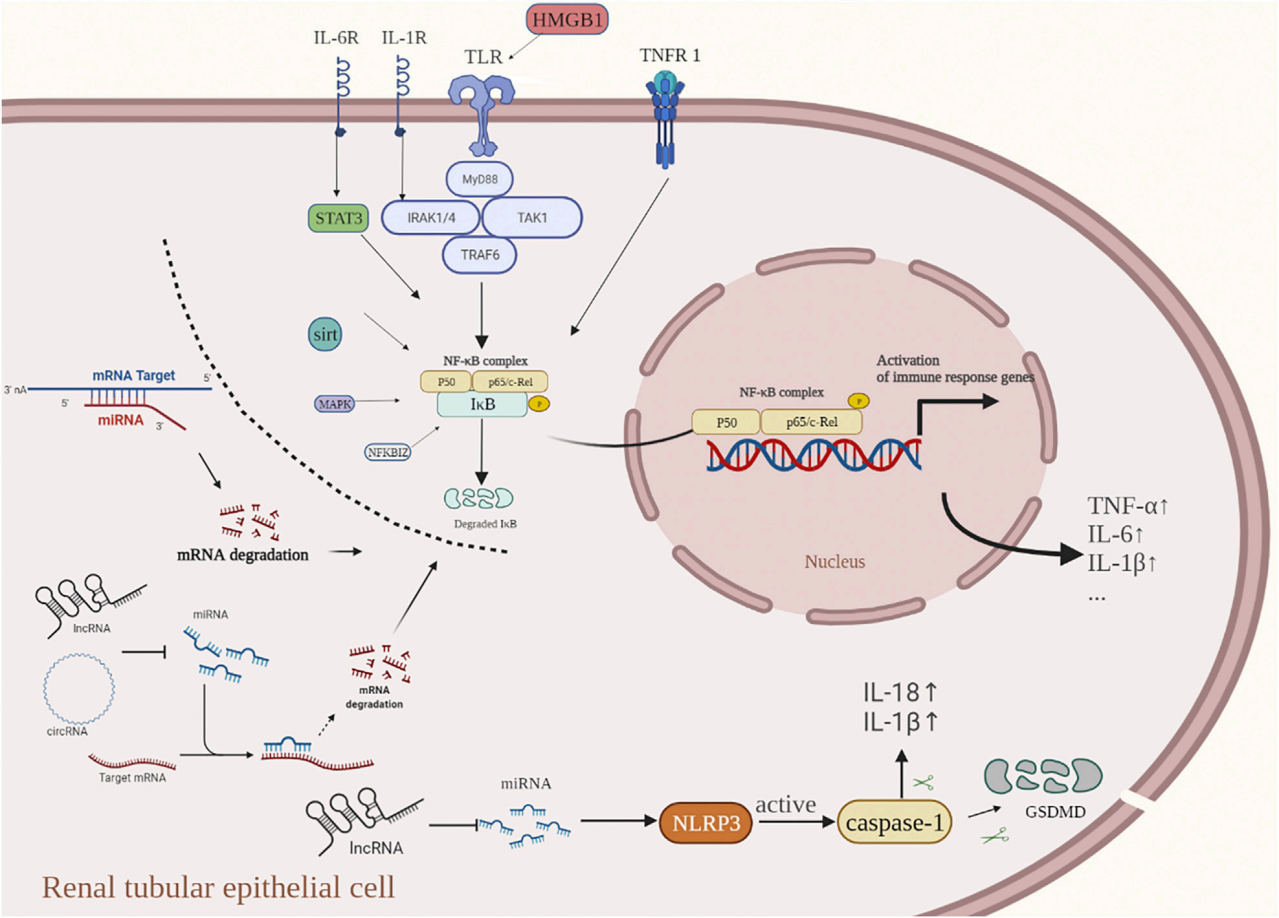
When cells are exposed to a variety of stimuli (inflammatory factors, bacterial infection, oxidative stress, etc.,). IκB flows through the degradation of the IKK phosphorylated proteasome, leading to abnormal NF-κB activation and nuclear translocation, thus promoting the transcription and translation of inflammation-related genes. The acute inflammatory cytokines TNF-α and IL-1β induce the activation of the NF-κB signaling pathway by activating the membrane receptors TNFR-1 and IL-1R, respectively. Activation of TLR4, a receptor on the renal tubular epithelial cell membrane, can form a complex of related factors to activate the NF-κB signal transduction pathway. SIRT1 reduces NF-κB activity and indirectly inhibits the expression of downstream inflammatory cytokines. Inflammasomes located in the cytoplasm can activate caspase-1 to release pro-inflammatory factors such as IL-1β and IL-18, and finally produce an inflammatory response. The current research suggests that ncRNAs play an important role in regulating the inflammatory response of AKI, and they are involved in regulating the NF-KB pathway and cell pyroptosis at the transcription level, thus regulating the inflammatory response. In the inflammatory response of AKI, ncRNAs usually work in the following ways: 1) miRNA directly targets the 3′-untranslated region (3′-UTR) of the target mRNA to inhibit transcription; 2) lncRNAs and circRNAs, commonly bind directly to miRNAs through competitive endogenous RNAs (ceRNAs) that promote up-regulation of target mRNA levels.

There is growing evidence that ncRNAs play an important role in AKI. However, the specific mechanisms by which ncRNAs regulate the expression of inflammation-related genes in AKI have not been fully elucidated. Clarifying the functional roles of ncRNAs and their intrinsic molecular mechanisms in regulating inflammatory responses will provide potential research strategies for developing targeted ncRNA gene therapies as an intervention for the inflammation-induced damage during AKI (Figure 3).

### Role of miRNAs in Inflammatory Response of Acute Kidney Injury

The most widely studied type of ncRNAs in AKI is the miRNAs. Many studies have shown that miRNAs play an essential role in kidney-related physiological and pathological processes by regulating the expression of post-transcriptional genes (Petejova et al., 2020). The results of large-scale high-throughput sequencing from patients and animals demonstrated that the miRNA expression profiles were dramatically changed in different types of AKI (Kumar et al., 2014; Kirita et al., 2019). The interaction network and mechanism between miRNAs and mRNAs, as well as their functions and effects on AKI inflammatory injury, are summarized in Table 1.

**TABLE 1:** Role of miRNAs in inflammatory response of AKI.

MiRNA	AKI model	Signaling pathways	Level
**Anti-inflammation**			
miRNA-125a-5p	Sepsis	TRAF6/NF-κB	↓
miRNA-129-5p	Sepsis	HMGB1/TLR2,4/NF-κB	↓
miRNA-20a	Sepsis	CXCL12/CXCR4/NF-κB	↓
miRNA-34b-3p	Sepsis	UBL4A/NF-κB	↓
miRNA-146b	Sepsis	IRAK1/NF-κB	↓
miRNA-93-5p	Sepsis	TXNIP/NLRP3	-
	Sepsis	KDM6B/H3K27me3/TNF-α	↓
miRNA-181d-5p	IRI	KLF6/NF-κB	↓
miRNA-21	IRI	PDCD4/NF-κB	↑
miRNA-500a-3p	Cisplatin	NF-κB	↓
**Proinflammation**			
miRNA-214-5p	Sepsis	GLP-1R/AMPK/NF-κB	↑
miRNA-376b	Sepsis	NF-κB/NFKBIZ	↓
miRNA-34b-5p	Sepsis	AQP2	↑
miRNA-494-3p	IRI	HTRA3/NF-κB	↑
miRNA-132-3p	Cisplatin	SIRT1/NF-κB	↑
miRNA-486-5p	Cisplatin	TLR4/NF-κB	↑

### MiRNAs Regulate the NF-κB Signaling Pathway

NF-κB plays a vital role in the expression of inflammation-related genes and is the central transcriptional regulator of the inflammatory response in AKI. Various miRNAs have been implicated in regulating the inflammatory response in AKI *via* NF-κB signaling. Yang et al. found that miRNA-125a-5p indirectly inhibited the TLR4/NF-κB signal transduction by targeting TRAF6, thus inhibiting the expression of pro-inflammatory cytokines and reducing LPS-induced kidney injury (Yang C. et al., 2022). Huang et al. found that up-regulation of miRNA-129-5p could reduce LPS-induced AKI by targeting HMGB1 to inhibit the TLR/NF-κB signaling pathway (Huang et al., 2020). Zhang et al. found that miRNA-20a could inactivate the NF-κB signaling pathway by targeting CXCL12, thereby inhibiting LPS-induced HK-2 cell injury (Zhang L. et al., 2020). Jiang et al. unveiled the therapeutic potential of miRNA-500a-3p in cisplatin-induced AKI, which could inhibit NF-κB-driven necrotic inflammation of renal epithelial cells (Jiang et al., 2019). A study showed that up-regulation of miRNA-181d-5p could ameliorate renal inflammatory injury by indirectly inhibiting NF-κB activity (Zhang Y. et al., 2020). Furthermore, continuous activation of NF-κB can promote the maturation of dendritic cells (O'Neill et al., 2013). In this regard, miRNA-21 plays a role in regulating inflammation by indirectly inhibiting NF-κB activity to prevent dendritic cell maturation and reduce IRI-induced kidney injury (Song et al., 2018).

In addition to playing an anti-inflammatory role, other miRNAs can also participate in the inflammatory response of AKI by activating the NF-κB signaling pathway. Lin et al. found that miRNA-486-5p can promote the cisplatin-induced acute inflammation response of renal tubular epithelial cells by directly targeting HAT1 *via* the TLR4/NF-κB pathway (Lin F. Y. et al., 2022). A study found that miRNA-494-3p could participate in the inflammatory response of tubular epithelial cells induced by hypoxia-reoxygenation *via* indirect activation of the NF-κB signaling pathway (Gong et al., 2021). Han et al. found that miRNA-132-3p indirectly activated the NF-κB pathway by negatively regulating SIRT1, thereby aggravating cisplatin-induced inflammatory response and apoptosis in renal tubular epithelial cells (Han et al., 2021). Guo et al. found that miRNA-214-5p could indirectly activate the NF-κB pathway by inhibiting AMPK, which aggravated the inflammatory damage in sepsis-induced AKI (Guo et al., 2021). In addition, different transcripts from the same miRNA source may have significantly different functions. For example, in AKI, miRNA-34b can regulate acute inflammation and apoptosis in sepsis by indirectly affecting the NF-κB signaling pathway; however, these effects can be both protective or damaging, respectively, dependent on whether its 3p- or 5p-chain structures are activated (He S. Y. et al., 2020; Zheng et al., 2021).

The nuclear transcription factor NF-κB can also regulate the inflammatory response in AKI by affecting the transcription of miRNAs. Liu et al. found that activation of NF-κB in AKI with sepsis could inhibit miRNA-376b transcription, indirectly relieving the targeted inhibition of NFKBIZ (a member of the IκB family of NF-κB inhibitory proteins) (Liu Z. et al., 2020). Unlike the classical IκB family, NFKBIZ inhibits the binding of miRNA-376b to DNA by binding to NF-κB through the carboxy-terminal anchored protein repeat domain, thereby reducing mRNA transcriptional activity (Göransson et al., 2009).

### Exosomal miRNAs Regulate Inflammatory Response

Exosomes are vesicles of 30–120 nm in size released into the extracellular space by the fusion of multivesicular bodies with the cell membrane (Kalluri and LeBleu, 2020). Their primary function is to transport substances or information, such as bioactive cytokines, ncRNAs, and membrane receptors between cells (Pegtel and Gould, 2019) to regulate the functional state of target cells (Cao J. et al., 2020). Targeting tubular epithelial cells with protective exosomal miRNAs is a potential therapeutic strategy for AKI (Cao et al., 2021). Chen et al. found that miRNA-93-5p in M2 macrophage-derived exosomes can indirectly alleviate LPS-induced tubular epithelial cell pyroptosis by inhibiting the NLRP3/IL-1β axis (Juan et al., 2021). He et al. found that exosomes containing miRNA-93-5p secreted by endothelial progenitor cells can alleviate sepsis-induced AKI by indirectly inhibiting the expression of TNF-α (He Z. et al., 2020). Zhang et al. found that human umbilical cord mesenchymal stem cell-derived exosome treatment indirectly caused the inhibition of NF-κB activity by up-regulating the level of miRNA-146b in renal tubular epithelial cells, thereby alleviating sepsis-induced AKI (Zhang R. et al., 2020).

### Role of lncRNAs in Inflammatory Response of Acute Kidney Injury

The abnormal expression of lncRNAs in AKI during inflammation is closely related to the inhibition or activation of inflammatory-related genes and signaling pathways. Enhancing or inhibiting gene transcription by interacting with transcription factors is the primary way lncRNAs participate in regulating inflammatory responses (Simion et al., 2020). Cytoplasmic-localized lncRNAs promote the upregulation of target mRNA levels by acting as competitive endogenous RNAs (ceRNAs) that bind directly to the miRNAs. However, it should be noted that nuclear lncRNAs can also regulate the biogenesis, distribution, and degradation of miRNAs at the transcriptional level to affect their function (Cheng et al., 2019; Ignarski et al., 2019). The roles and mechanism of lncRNAs as ceRNAs in AKI-related inflammatory responses are summarized in Table 2.

**TABLE 2:** Role of lncRNAs in inflammatory response of AKI.

LncRNA	AKI model	Signaling Pathways	Level
**Anti-inflammation**			
CCAT1	Sepsis	miRNA-155/SIRT1/NF-κB	↓
HOXA-AS2	Sepsis	miRNA-106b-5p/SIRT1/NF-κB	↓
XIST	Sepsis	miRNA-155-5p/WWC1	↓
CASC9	Sepsis	miRNA-424-5p/TXNIP	↓
**Proinflammation**			
SNHG14	Sepsis	miRNA-93/IRAK4/NF-*κ*B	↑
	Sepsis	miRNA-495-3p/HIPK1	↑
	IRI	miRNA-124-3p/MMP2	
KCNQ1OT1	Sepsis	miRNA-212-3p/MAPK1/NF-κB	↑
	IRI	miRNA-204-5p/NLRP3	↑
DLX6-AS1	Sepsis	miRNA-223-3p/NLRP3	↑
MEG3	Sepsis	miRNA-18a-3p/GSDMD	↑
PVT1	Sepsis	miRNA-20a-5p/NLRP3	↑
SNHG5	Sepsis	miRNA-374a-3p/TLR4/NF-κB	↑
NEAT1	Sepsis	miRNA-22-3p/NF-κB	↑
	Sepsis	miRNA-93-5p/TXNIP	↑
MALAT1	Sepsis	miRNA-135b-5p/NLRP3	↑
NORAD	Sepsis	miRNA-577/GOLPH3	↑

### Interaction Between the lncRNA/miRNA Axis and NF-κB Signaling Pathway

Nuclear transcription regulation is an essential function of lncRNAs, and its influence on the NF-κB pathway is most important. LncRNAs can affect the expression of inflammatory factors by acting on the activation of NF-κB and related signaling pathways. A study on the up-regulation of SNHG14 in LPS-induced AKI indicated that the SNHG14/miRNA-495-3p axis promoted the expression of inflammatory cytokines by activating NF-κB signaling (Yang N. et al., 2021). The activated membrane receptor IL-1R mediates the activation of the NF-κB pathway through IRAK4, leading to the transcription of downstream inflammatory cytokines (Verstrepen et al., 2008). IL-6R can activate the nuclear transcription factor STAT3 and nuclear translocation, thus promoting the expression of the corresponding effector genes (Nechemia-Arbely et al., 2008). It has been found that SNHG14 promotes LPS-induced inflammatory injury in renal tubular epithelial cells by targeting miRNA-93 to regulate the IRAK4/NF-κB and IL-6R/STAT3 signaling pathways (Shi et al., 2021). KCNQ1OT1 can indirectly activate the MAPK1/NF-κB signaling pathway by competitively binding miRNA-212-3p, thus aggravating sepsis-induced AKI (Wang H. et al., 2021). Down-regulating lncRNA involved in the activation of NF-κB could reduce the inflammatory damage of AKI. Wang et al. found that down-regulation of SNHG5 reduced sepsis-induced AKI *via* inhibition of the TLR4/NF-κB signaling pathway by targeting miRNA-374a-3p (Wang M. et al., 2021). Feng et al. found that knock-down of NEAT1 alleviated sepsis-induced AKI by enhancing the inhibition of NF-κB *via* miRNA-22-3p (Feng et al., 2020).

Some lncRNAs, such as CCAT1 and HOXA-AS2, have protective effects, as they can reduce the inflammatory response in AKI by inhibiting the NF-κB signaling pathway. A study in LPS-induced AKI showed that up-regulation of CCAT1 could restore the inhibitory effect of SIRT1 on NF-κB by targeting miRNA-155. This, in turn, reduced the LPS-induced inflammatory response and apoptosis of renal tubular epithelial cells (Lu et al., 2020). Another study showed that HOXA-AS2 has a protective effect in sepsis-induced AKI, as it reduces the inflammatory damage of tubular epithelial cells by targeting the inhibition of miRNA-106b-5p and blocking the activation of the NF-κB pathway (Wu et al., 2020). Nevertheless, the function of some lncRNAs in AKI is controversial. For instance, one study indicated that CRNDE protects renal tubular epithelial cells from sepsis-induced inflammatory injury by targeting miRNA-181a-5 (Wang J. et al., 2020). In contrast, another study showed that knock-down of CRNDE can alleviate sepsis-induced tubular epithelial cell injury in AKI by inhibiting the TLR3/NF-κB pathway (Sun et al., 2019).

### LncRNA/miRNA Axis Regulates Cellular Pyroptosis and Inflammatory Responses

When cell pyroptosis occurs, inflammation can be caused by the release of pro-inflammatory cytokines IL-1β and IL-18. The active N-terminal of GSDMD is the “molecular switch” needed to open the cell membrane pores and release inflammatory factors in the classical cell pyroptosis pathway (Kim et al., 2019). LncRNAs such as MEG3, DLX6-AS1, PVT1, KCNQ1OT1, and MALAT1 are related to cell pyroptosis and its mediated inflammatory response. Up-regulation of MEG3 promotes the tubular epithelial cell pyroptosis and inflammatory response in LPS-induced AKI by relieving the inhibitory effect of miRNA-18a-3p on GSDMD (Deng J. et al., 2021). Inhibiting the pyroptosis-inducing lncRNAs can also help to alleviate the inflammatory injury in AKI. Tan et al. found that knock-down of lncRNA DLX6-AS1 inhibits pyroptosis and inflammatory responses in HK-2 cells in LPS-induced AKI by restoring the inhibitory effect of miRNA-223-3p on NLRP3 (Tan et al., 2020). Deng et al. found that knock-down of lncRNA PVT1 inhibits LPS-induced cell pyroptosis and inflammatory response by targeting the miRNA-20a-5p/NLRP3 signaling pathway (Deng L.-T. et al., 2021). Wang et al. found that silencing KCNQ1OT1, a ceRNA that promotes inflammatory injury in AKI, could indirectly inhibit NLRP3 inflammasomes through targeted activation of miRNA-204-5p to ameliorate IRI-induced inflammatory injury in renal tubular epithelial cells (Wang J. et al., 2021). Huang et al. found in LPS-induced renal tubular epithelial cells that knock-down of MALAT1, an inflammatory response regulator and biomarker for patients with sepsis (Ma T. et al., 2021), indirectly inhibited NLRP3-mediated pyroptosis and inflammatory response by releasing miRNA-135b-5p (Huang and Xu, 2021).

### Other lncRNA-miRNA Regulatory Axes

he ceRNA network consisting of lncRNAs, miRNAs, and mRNAs, is involved in the pathogenesis of AKI by altering the expression pattern of inflammation-related genes (Braga et al., 2020). For instance, the lncRNA NEAT1 is a novel inflammatory regulator that can regulate TXNIP expression acting as a sponge for miRNA-93-5p, thus promoting LPS-induced inflammatory injury in renal tubular epithelial cells (Yang J. et al., 2021). Inhibition of lncRNAs with pro-inflammatory properties can attenuate renal inflammatory injury in AKI. Furthermore, a study on the upregulation of SNHG14 in ischemic-hypoxic AKI showed that knock-down of SNHG14 ameliorated inflammatory injury in renal tubular epithelial cells by targeting miRNA-124-3p/MMP2(Xue et al., 2021). In addition to exerting pro-inflammatory effects, the lncRNA-miRNA axis can also suppress the acute renal inflammatory response. Wang et al. found that XIST acts as a ceRNA for miRNA-155-5p to suppress inflammation and apoptosis, thereby attenuating sepsis-induced AKI(Wang and Cao, 2022). Furthermore, Fan et al. found that the CASC9 could attenuate LPS-induced acute renal inflammatory injury *via* the miRNA-424-5p/TXNIP axis (Fan et al., 2021). The latest research showed that NORAD knockdown alleviated kidney injury in mice and decreased the inflammatory response and apoptosis of LPS-stimulated HK-2 cells *via* the miRNA-577/GOLPH3 axis (Xie et al., 2022).

### Role of circRNAs in the Inflammatory Response of Acute Kidney Injury

Based on high-throughput sequencing technology and bioinformatics analyses, many studies have revealed differential expression profiles of circRNAs in AKI (Li et al., 2019). Similar to lncRNAs, circRNAs mainly participate in regulating the inflammatory response of AKI by forming a circRNA-miRNA-mRNA action network (Jin et al., 2020). Some circRNAs have been shown to inhibit or promote the inflammatory response in AKI by targeted inhibition of miRNAs (Table 3).

**TABLE 3:** Role of circRNAs in inflammatory response of AKI.

CircRNA	AKI model	Signaling Pathways	Level
**Anti-inflammation**			
VMA21	Sepsis	miRNA-9-3p/SMG1/NF-κB	↓
	Sepsis	miRNA-7-5p/PPARA	↓
TTC3	Sepsis	miRNA-148a/RCAN2	↓
0,114,427	Cisplatin	miRNA-494/ATF3/NF-κB	↑
**Proinflammation**			
BNIP3L	Sepsis	miRNA-370-3p/MyD88/NF-κB	↑
TLK1	Sepsis	miRNA-106a-5p/HMGB1/NF-κB	↑
0,114,427	Sepsis	miRNA-495-3p/TRAF6/NF-κB	↑
HIPK3	Sepsis	miRNA-338/FOXA1	↑
0,023,404	IRI	miRNA-136/IL-6R	↑
0,000,943	IRI	miRNA-377-3p/EGR2	↑

### Promoting Inflammatory Response

A study found that the circRNA BNIP3L can promote LPS-induced apoptosis and the inflammatory response by the miRNA-370-3p/MyD88 axis in HK-2 cells (Zhou et al., 2021a). Another study showed that the circRNA TLK1 indirectly enhances HMGB1 expression by sponging miRNA-106a-5p, thus aggravating the sepsis-induced inflammatory response in renal tubular epithelial cells (Xu H.-P. et al., 2021). A study on the up-regulation of circ_0114427 in sepsis in AKI showed that it promoted the apoptosis of renal tubular epithelial cells and inflammatory response by targeting miRNA-495-3p to activate the TRAF6/NF-κB signaling pathway (Xu et al., 2022). Additionally, it has been shown that circRNA HIPK3 aggravates LPS-induced inflammation *via* the miRNA-338/FOXA1 axis (Lu et al., 2022). CircRNA can also play a pro-inflammatory role in non-infectious AKI. A study has shown that circ_0023404 can participate in hypoxia-reoxygenation-induced inflammatory injury in HK-2 cells by sponging miRNA-136 and activating IL-6R (Xu Y. et al., 2021). The latest study suggested that circ_0000943 regulated the expression of EGR2 by sponging miRNA-377-3p to aggravate inflammation in renal IRI (Huang et al., 2022).

### Inhibition of Inflammatory Response

Two different studies have shown that the circRNA VMA21 can simultaneously serve as a sponge for miRNA-9-3p and miRNA-7-5p. Furthermore, up-regulation of circRNA VMA21 can alleviate sepsis-related AKI by inhibiting the expression of these two miRNAs (Shi et al., 2020; Wang et al., 2022). Another study on the down-regulation of circRNA TTC3 in sepsis-related AKI showed that up-regulation of circRNA TTC3 can alleviate the sepsis-induced inflammatory response in renal tubular epithelial cells by targeted inhibition of miRNA-148a (Ma X. et al., 2021). Moreover, the pro-inflammatory property of cisplatin is potentially destructive (McSweeney et al., 2021). Discovering effective circRNA and studying their intrinsic molecular regulation pathways to reduce kidney inflammation is one of the most promising methods to determine the target of early intervention with cisplatin-induced AKI (Holditch et al., 2019). A recent study found that circRNA 0114427 could release the inhibition of miRNA-494 on ATF3 by acting as a miRNA sponge, while ATF3 could block the activation of NF-κB to reduce the cisplatin-induced inflammatory response in AKI (Cao Y. et al., 2020).

Our current understanding of the function and role of circRNAs is still in its infancy, and further research is needed to clarify the specific regulatory mechanism of circRNAs in the inflammation of AKI. However, the study of circRNAs as a therapeutic target for AKI will undoubtedly be a hot topic in the future.

### The Challenge of Studying

Understanding the role of ncRNAs in AKI inflammation can provide potential research directions and strategies for the development of ncRNA-targeted gene therapy as an intervention for AKI inflammation injury. However, studies into the regulation of inflammation by ncRNAs have mainly been conducted in animal experiments, with a huge gap to fill before clinical trials. Therefore, extensive research is needed to further explore the clinical value of ncRNAs. In addition, identifying homologous ncRNAs between experimental animals and humans is a tremendous work in itself (Kulkarni et al., 2021). Most ncRNAs are not limited to a certain cell type or tissue but ubiquitously expressed (Matsui and Corey, 2017). Therefore, regulation of specific ncRNAs may lead to so-called “off-target” effects in distant organs. This dilemma is reflected in the fact that only a few ongoing clinical studies involve miRNA therapy (Winkle et al., 2021). Elucidation of the role of specific ncRNAs is thus a prerequisite for RNA-based targeted therapy for specific diseases. Tissue/cell specificity can also be achieved by coupling ncRNAs to tissue-specific antibodies and/or peptides, thereby reducing the effects of off-targeting (Winkle et al., 2021). Besides, ncRNAs are also involved in various pathological processes of AKI, in addition to inflammatory reactions, and uncertainty remains as to whether ncRNAs can affect the overall function of the kidney by regulating a specific reaction. Despite recent studies reporting promising therapeutic effects of ncRNAs, their therapeutic application in targeting molecules in AKI is still a long way off.

## Conclusion and Prospects

Inflammation is an extremely important part of the development of AKI, and ncRNAs involved in the regulation of gene expression play an important role in this process. At present, research into the role of ncRNAs in regulating the inflammatory response in AKI has mainly focused on miRNAs and lncRNAs, though elucidating the mechanism of circRNAs has been in full swing. The mechanistic role of ncRNAs in the inflammatory response of AKI is gradually being uncovered, with multi-target inhibition or overexpression shown to be effective at reducing AKI-related inflammation in animal or cell models. Inhibition or rescue of these dysregulated ncRNAs *in vivo* represents a fascinating new dimension in therapeutic regulation of the inflammatory response in AKI. Technically, *in vivo* anti-sense oligonucleotide therapy for targeted regulation of ncRNAs is feasible. However, many challenges must be overcome from an experimental research perspective before clinical translation, including careful evaluation of potential off-target effects caused by low ncRNA specificity. Future work is needed to address the shortcomings of the current research and increase our understanding of the regulatory mechanism of ncRNAs in AKI. A better understanding of the mechanisms behind ncRNAs will potentially result in its safe and effective use as a precise treatment for patients with AKI.

## References

[B1] Ali T. Grote P. (2020). Beyond the RNA-Dependent Function of LncRNA Genes. Elife 9. 10.7554/eLife.60583 PMC7584451 33095159

[B2] Andrade-Oliveira V. Foresto-Neto O. Watanabe I. K. M. Zatz R. Câmara N. O. S. (2019). Inflammation in Renal Diseases: New and Old Players. Front. Pharmacol. 10, 1192. 10.3389/fphar.2019.01192 31649546 PMC6792167

[B3] Baek J.-H. (2019). The Impact of Versatile Macrophage Functions on Acute Kidney Injury and its Outcomes. Front. Physiol. 10. 10.3389/fphys.2019.01016 PMC6691123 31447703

[B4] Bejoy J. Qian E. S. Woodard L. E. (2022). Tissue Culture Models of AKI: From Tubule Cells to Human Kidney Organoids. J. Am. Soc. Nephrol. 33 (3), 487–501. 10.1681/asn.2021050693 35031569 PMC8975068

[B5] Braga E. A. Fridman M. V. Moscovtsev A. A. Filippova E. A. Dmitriev A. A. Kushlinskii N. E. (2020). LncRNAs in Ovarian Cancer Progression, Metastasis, and Main Pathways: ceRNA and Alternative Mechanisms. Ijms 21 (22), 8855. 10.3390/ijms21228855 PMC7700431 33238475

[B6] Bridges M. C. Daulagala A. C. Kourtidis A. (2021). LNCcation: lncRNA Localization and Function. J. Cell Biol. 220 (2). 10.1083/jcb.202009045 PMC7816648 33464299

[B7] Cao J.-Y. Wang B. Tang T.-T. Wen Y. Li Z.-L. Feng S.-T. (2021). Exosomal miR-125b-5p Deriving from Mesenchymal Stem Cells Promotes Tubular Repair by Suppression of P53 in Ischemic Acute Kidney Injury. Theranostics 11 (11), 5248–5266. 10.7150/thno.54550 33859745 PMC8039965

[B8] Cao J. Wang B. Tang T. Lv L. Ding Z. Li Z. (2020a). Three-Dimensional Culture of MSCs Produces Exosomes with Improved Yield and Enhanced Therapeutic Efficacy for Cisplatin-Induced Acute Kidney Injury. Stem Cell Res. Ther. 11 (1), 206. 10.1186/s13287-020-01719-2 32460853 PMC7251891

[B9] Cao Y. Mi X. Zhang D. Wang Z. Zuo Y. Tang W. (2020b). Transcriptome Sequencing of Circular RNA Reveals a Novel Circular RNA-Has_circ_0114427 in the Regulation of Inflammation in Acute Kidney Injury. Clin. Sci. (Lond) 134 (2), 139–154. 10.1042/cs20190990 31930399

[B10] Carling D. (2017). AMPK Signalling in Health and Disease. Curr. Opin. Cell Biol. 45, 31–37. 10.1016/j.ceb.2017.01.005 28232179

[B11] Cech T. R. Steitz J. A. (2014). The Noncoding RNA Revolution-Trashing Old Rules to Forge New Ones. Cell 157 (1), 77–94. 10.1016/j.cell.2014.03.008 24679528

[B12] Chen L.-L. (2016). Linking Long Noncoding RNA Localization and Function. Trends Biochem. Sci. 41 (9), 761–772. 10.1016/j.tibs.2016.07.003 27499234

[B13] Chen L. Wang C. Sun H. Wang J. Liang Y. Wang Y. (2021a). The Bioinformatics Toolbox for circRNA Discovery and Analysis. Brief. Bioinform 22 (2), 1706–1728. 10.1093/bib/bbaa001 32103237 PMC7986655

[B14] Chen X.-T. Li Z.-W. Zhao X. Li M.-L. Hou P.-F. Chu S.-F. (2021b). Role of Circular RNA in Kidney-Related Diseases. Front. Pharmacol. 12, 615882. 10.3389/fphar.2021.615882 33776764 PMC7990792

[B15] Chen X. Yang T. Wang W. Xi W. Zhang T. Li Q. (2019). Circular RNAs in Immune Responses and Immune Diseases. Theranostics 9 (2), 588–607. 10.7150/thno.29678 30809295 PMC6376182

[B16] Chen Y. Jin S. Teng X. Hu Z. Zhang Z. Qiu X. (2018). Hydrogen Sulfide Attenuates LPS-Induced Acute Kidney Injury by Inhibiting Inflammation and Oxidative Stress. Oxidative Med. Cell. Longev. 2018, 1–10. 10.1155/2018/6717212 PMC5831990 29636853

[B17] Cheng W. Li X.-W. Xiao Y.-Q. Duan S.-B. (2019). Non-coding RNA-Associated ceRNA Networks in a New Contrast-Induced Acute Kidney Injury Rat Model. Mol. Ther. - Nucleic Acids 17, 102–112. 10.1016/j.omtn.2019.05.011 31234008 PMC6595412

[B18] Deng B. Lin Y. Chen Y. Ma S. Cai Q. Wang W. (2021a). Plasmacytoid Dendritic Cells Promote Acute Kidney Injury by Producing Interferon-α. Cell Mol. Immunol. 18 (1), 219–229. 10.1038/s41423-019-0343-9 31900458 PMC7852581

[B19] Deng J. Tan W. Luo Q. Lin L. Zheng L. Yang J. (2021b). Long Non-Coding RNA MEG3 Promotes Renal Tubular Epithelial Cell Pyroptosis by Regulating the miR-18a-3p/GSDMD Pathway in Lipopolysaccharide-Induced Acute Kidney Injury. Front. Physiol. 12, 663216. 10.3389/fphys.2021.663216 34012408 PMC8128073

[B20] Deng L.-T. Wang Q.-L. Yu C. Gao M. (2021c). lncRNA PVT1 Modulates NLRP3-Mediated Pyroptosis in Septic Acute Kidney Injury by Targeting miR-20a-5p. Mol. Med. Rep. 23 (4). 10.3892/mmr.2021.11910 33576456

[B21] Diederichs S. (2014). The Four Dimensions of Noncoding RNA Conservation. Trends Genet. 30 (4), 121–123. 10.1016/j.tig.2014.01.004 24613441

[B22] Esteller M. (2011). Non-Coding RNAs in Human Disease. Nat. Rev. Genet. 12 (12), 861–874. 10.1038/nrg3074 22094949

[B23] Fan H.-P. Zhu Z.-X. Xu J.-J. Li Y.-T. Guo C.-W. Yan H. (2021). The lncRNA CASC9 Alleviates Lipopolysaccharide-Induced Acute Kidney Injury by Regulating the miR-424-5p/TXNIP Pathway. J. Int. Med. Res. 49 (8), 030006052110374. 10.1177/03000605211037495 PMC8381429 34407684

[B24] Fani F. Regolisti G. Delsante M. Cantaluppi V. Castellano G. Gesualdo L. (2018). Recent Advances in the Pathogenetic Mechanisms of Sepsis-Associated Acute Kidney Injury. J. Nephrol. 31 (3), 351–359. 10.1007/s40620-017-0452-4 29273917

[B25] Feng Y. Liu J. Wu R. Yang P. Ye Z. Song F. (2020). NEAT1 Aggravates Sepsis-Induced Acute Kidney Injury by Sponging miR-22-3p. Open Med. (Wars) 15 (1), 333–342. 10.1515/med-2020-0401 33335994 PMC7712373

[B26] Ferrè F. Colantoni A. Helmer-Citterich M. (2016). Revealing Protein-lncRNA Interaction. Brief. Bioinform 17 (1), 106–116. 10.1093/bib/bbv031 26041786 PMC4719072

[B27] Gao Z. Lu L. Chen X. (2021). Release of HMGB1 in Podocytes Exacerbates Lipopolysaccharide-Induced Acute Kidney Injury. Mediat. Inflamm. 2021, 1–10. 10.1155/2021/5220226 PMC8490059 34616232

[B28] Giménez-Arnau A. M. de Montjoye L. Asero R. Cugno M. Kulthanan K. Yanase Y. (2021). The Pathogenesis of Chronic Spontaneous Urticaria: The Role of Infiltrating Cells. J. Allergy Clin. Immunol. Pract. 9 (6), 2195–2208. 10.1016/j.jaip.2021.03.033 33823316

[B29] Glass C. K. Saijo K. Winner B. Marchetto M. C. Gage F. H. (2010). Mechanisms Underlying Inflammation in Neurodegeneration. Cell 140 (6), 918–934. 10.1016/j.cell.2010.02.016 20303880 PMC2873093

[B30] Godin M. Murray P. Mehta R. L. (2015). Clinical Approach to the Patient with AKI and Sepsis. Seminars Nephrol. 35 (1), 12–22. 10.1016/j.semnephrol.2015.01.003 PMC5617729 25795496

[B31] Gong Q. Shen Z.-m. Sheng Z. Jiang S. Ge S.-l. (2021). Hsa-miR-494-3p Attenuates Gene HtrA3 Transcription to Increase Inflammatory Response in Hypoxia/Reoxygenation HK2 Cells. Sci. Rep. 11 (1), 1665. 10.1038/s41598-021-81113-x 33462352 PMC7814133

[B32] Göransson M. Andersson M. K. Forni C. Ståhlberg A. Andersson C. Olofsson A. (2009). The Myxoid Liposarcoma FUS-DDIT3 Fusion Oncoprotein Deregulates NF-Κb Target Genes by Interaction with NFKBIZ. Oncogene 28 (2), 270–278. 10.1038/onc.2008.378 18850010

[B33] Guo C. Ye F. X. Jian Y. H. Liu C. H. Tu Z. H. Yang D. P. (2021). MicroRNA‐214‐5p Aggravates Sepsis‐Related Acute Kidney Injury in Mice. Drug Dev. Res. 83, 339–350. 10.1002/ddr.21863 34370322

[B34] Habib R. (2021). Multifaceted Roles of Toll-Like Receptors in Acute Kidney Injury. Heliyon 7 (3), e06441. 10.1016/j.heliyon.2021.e06441 33732942 PMC7944035

[B35] Hamroun A. Lenain R. Bigna J. J. Speyer E. Bui L. Chamley P. (2019). Prevention of Cisplatin-Induced Acute Kidney Injury: A Systematic Review and Meta-Analysis. Drugs 79 (14), 1567–1582. 10.1007/s40265-019-01182-1 31429065

[B36] Han S. J. Lee H. T. (2019). Mechanisms and Therapeutic Targets of Ischemic Acute Kidney Injury. Kidney Res. Clin. Pract. 38 (4), 427–440. 10.23876/j.krcp.19.062 31537053 PMC6913588

[B37] Han S. Lin F. Ruan Y. Zhao S. Yuan R. Ning J. (2021). miR-132-3p Promotes the Cisplatin-Induced Apoptosis and Inflammatory Response of Renal Tubular Epithelial Cells by Targeting SIRT1 via the NF-Κb Pathway. Int. Immunopharmacol. 99, 108022. 10.1016/j.intimp.2021.108022 34339961

[B38] He L. Wei Q. Liu J. Yi M. Liu Y. Liu H. (2017). AKI on CKD: Heightened Injury, Suppressed Repair, and the Underlying Mechanisms. Kidney Int. 92 (5), 1071–1083. 10.1016/j.kint.2017.06.030 28890325 PMC5683166

[B39] He S. Y. Wang G. Pei Y. H. Zhu H. P. (2020a). miR‐34b‐3p Protects Against Acute Kidney Injury in Sepsis Mice via Targeting Ubiquitin‐Like Protein 4A. Kaohsiung J. Med. Sci. 36 (10), 817–824. 10.1002/kjm2.12255 32609950 PMC11896296

[B40] He Z. Wang H. Yue L. (2020b). Endothelial Progenitor Cells-Secreted Extracellular Vesicles Containing microRNA-93-5p Confer Protection Against Sepsis-Induced Acute Kidney Injury via the KDM6B/H3K27me3/TNF-α Axis. Exp. Cell Res. 395 (2), 112173. 10.1016/j.yexcr.2020.112173 32679234

[B41] Hepokoski M. Wang J. Li K. Li Y. Gupta P. Mai T. (2021). Altered Lung Metabolism and Mitochondrial DAMPs in Lung Injury Due to Acute Kidney Injury. Am. J. Physiology-Lung Cell. Mol. Physiology 320 (5), L821–l831. 10.1152/ajplung.00578.2020 PMC8174821 33565357

[B42] Holditch S. J. Brown C. N. Lombardi A. M. Nguyen K. N. Edelstein C. L. (2019). Recent Advances in Models, Mechanisms, Biomarkers, and Interventions in Cisplatin-Induced Acute Kidney Injury. Ijms 20 (12), 3011. 10.3390/ijms20123011 PMC6627318 31226747

[B43] Holdsworth S. R. Gan P.-Y. (2015). Cytokines: Names and Numbers You Should Care about. Clin. J. Am. Soc. Nephrol. 10 (12), 2243–2254. 10.2215/cjn.07590714 25941193 PMC4670773

[B44] Hoste E. A. J. Bagshaw S. M. Bellomo R. Cely C. M. Colman R. Cruz D. N. (2015). Epidemiology of Acute Kidney Injury in Critically Ill Patients: The Multinational AKI-EPI Study. Intensive Care Med. 41 (8), 1411–1423. 10.1007/s00134-015-3934-7 26162677

[B45] Hu J. Qiao J. Yu Q. Liu B. Zhen J. Liu Y. (2021a). Role of SIK1 in the Transition of Acute Kidney Injury into Chronic Kidney Disease. J. Transl. Med. 19 (1), 69. 10.1186/s12967-021-02717-5 33588892 PMC7885408

[B46] Hu Q. Lyon C. J. Fletcher J. K. Tang W. Wan M. Hu T. Y. (2021b). Extracellular Vesicle Activities Regulating Macrophage- and Tissue-Mediated Injury and Repair Responses. Acta Pharm. Sin. B 11 (6), 1493–1512. 10.1016/j.apsb.2020.12.014 34221864 PMC8245807

[B47] Huang J. Xu C. (2021). LncRNA MALAT1-Deficiency Restrains Lipopolysaccharide (LPS)-Induced Pyroptotic Cell Death and Inflammation in HK-2 Cells by Releasing microRNA-135b-5p. Ren. Fail. 43 (1), 1288–1297. 10.1080/0886022x.2021.1974037 34503385 PMC8439250

[B48] Huang T. Gao Y. Cao Y. Wang Q. Dong Z. (2022). Downregulation of Mmu_circ_0000943 Ameliorates Renal Ischemia Reperfusion-Triggered Inflammation and Oxidative Stress via Regulating Mmu-miR-377-3p/Egr2 Axis. Int. Immunopharmacol. 106, 108614. 10.1016/j.intimp.2022.108614 35168080

[B49] Huang X. Hou X. Chuan L. Wei S. Wang J. Yang X. (2020). miR-129-5p Alleviates LPS-Induced Acute Kidney Injury via Targeting HMGB1/TLRs/NF-kappaB Pathway. Int. Immunopharmacol. 89 (Pt A), 107016. 10.1016/j.intimp.2020.107016 33039954

[B50] Ignarski M. Islam R. Müller R.-U. (2019). Long Non-Coding RNAs in Kidney Disease. Ijms 20 (13), 3276. 10.3390/ijms20133276 PMC6650856 31277300

[B51] Jiang L. Liu X.-Q. Ma Q. Yang Q. Gao L. Li H.-D. (2019). hsa‐miR‐500a‐3P Alleviates Kidney Injury by Targeting MLKL‐Mediated Necroptosis in Renal Epithelial Cells. FASEB J. 33 (3), 3523–3535. 10.1096/fj.201801711R 30365367

[B52] Jiang L. Zhu J. (2020). Review of MiRNA-Disease Association Prediction. Cpps 21 (11), 1044–1053. 10.2174/1389203721666200210102751 32039677

[B53] Jiao B. An C. Du H. Tran M. Wang P. Zhou D. (2021a). STAT6 Deficiency Attenuates Myeloid Fibroblast Activation and Macrophage Polarization in Experimental Folic Acid Nephropathy. Cells 10 (11), 3057. 10.3390/cells10113057 34831280 PMC8623460

[B54] Jiao B. An C. Tran M. Du H. Wang P. Zhou D. (2021b). Pharmacological Inhibition of STAT6 Ameliorates Myeloid Fibroblast Activation and Alternative Macrophage Polarization in Renal Fibrosis. Front. Immunol. 12, 735014. 10.3389/fimmu.2021.735014 34512669 PMC8426438

[B55] Jiao F. Gong Z. (2020). The Beneficial Roles of SIRT1 in Neuroinflammation-Related Diseases. Oxidative Med. Cell. Longev. 2020, 1–19. 10.1155/2020/6782872 PMC7519200 33014276

[B56] Jin J. Sun H. Shi C. Yang H. Wu Y. Li W. (2020). Circular RNA in Renal Diseases. J. Cell Mol. Med. 24 (12), 6523–6533. 10.1111/jcmm.15295 32333642 PMC7299708

[B57] Juan C. X. Mao Y. Cao Q. Chen Y. Zhou L. B. Li S. (2021). Exosome‐Mediated Pyroptosis of miR‐93‐TXNIP‐NLRP3 Leads to Functional Difference Between M1 and M2 Macrophages in Sepsis‐Induced Acute Kidney Injury. J. Cell Mol. Med. 25 (10), 4786–4799. 10.1111/jcmm.16449 33745232 PMC8107088

[B58] Kalluri R. LeBleu V. S. (2020). The Biology, Function, and Biomedical Applications of Exosomes. Science 367 (6478). 10.1126/science.aau6977 PMC7717626 32029601

[B59] Kaushal G. P. Shah S. V. (2016). Autophagy in Acute Kidney Injury. Kidney Int. 89 (4), 779–791. 10.1016/j.kint.2015.11.021 26924060 PMC4801755

[B60] Kher A. Kher V. (2020). Prevention and Therapy of AKI in Asia: A Big Challenge. Seminars Nephrol. 40 (5), 477–488. 10.1016/j.semnephrol.2020.08.004 33334461

[B61] Kim I. Kwak H. Lee H. K. Hyun S. Jeong S. (2012). β-Catenin Recognizes a Specific RNA Motif in the Cyclooxygenase-2 mRNA 3′-UTR and Interacts with HuR in Colon Cancer Cells. Nucleic Acids Res. 40 (14), 6863–6872. 10.1093/nar/gks331 22544606 PMC3413138

[B62] Kim Y. G. Kim S.-M. Kim K.-P. Lee S.-H. Moon J.-Y. (2019). The Role of Inflammasome-Dependent and Inflammasome-Independent NLRP3 in the Kidney. Cells 8 (11), 1389. 10.3390/cells8111389 PMC6912448 31694192

[B63] Kirita Y. Chang-Panesso M. Humphreys B. D. (2019). Recent Insights into Kidney Injury and Repair From Transcriptomic Analyses. Nephron 143 (3), 162–165. 10.1159/000500638 31112966 PMC6821561

[B64] Kopp F. Mendell J. T. (2018). Functional Classification and Experimental Dissection of Long Noncoding RNAs. Cell 172 (3), 393–407. 10.1016/j.cell.2018.01.011 29373828 PMC5978744

[B65] Kormann R. Kavvadas P. Placier S. Vandermeersch S. Dorison A. Dussaule J.-C. (2020). Periostin Promotes Cell Proliferation and Macrophage Polarization to Drive Repair After AKI. J. Am. Soc. Nephrol. 31 (1), 85–100. 10.1681/asn.2019020113 31690575 PMC6935011

[B66] Kristensen L. S. Andersen M. S. Stagsted L. V. W. Ebbesen K. K. Hansen T. B. Kjems J. (2019). The Biogenesis, Biology and Characterization of Circular RNAs. Nat. Rev. Genet. 20 (11), 675–691. 10.1038/s41576-019-0158-7 31395983

[B67] Kulkarni J. A. Witzigmann D. Thomson S. B. Chen S. Leavitt B. R. Cullis P. R. (2021). The Current Landscape of Nucleic Acid Therapeutics. Nat. Nanotechnol. 16 (6), 630–643. 10.1038/s41565-021-00898-0 34059811

[B68] Kumar S. Liu J. McMahon A. P. (2014). Defining the Acute Kidney Injury and Repair Transcriptome. Seminars Nephrol. 34 (4), 404–417. 10.1016/j.semnephrol.2014.06.007 PMC4163949 25217269

[B69] Kurts C. Panzer U. Anders H.-J. Rees A. J. (2013). The Immune System and Kidney Disease: Basic Concepts and Clinical Implications. Nat. Rev. Immunol. 13 (10), 738–753. 10.1038/nri3523 24037418

[B70] Kusmartsev S. (2021). Acute Kidney Injury-Induced Systemic Inflammation and Risk of Kidney Cancer Formation. Cancer Res. 81 (10), 2584–2585. 10.1158/0008-5472.Can-21-0807 33999840

[B71] Lan R. Geng H. Singha P. K. Saikumar P. Bottinger E. P. Weinberg J. M. (2016). Mitochondrial Pathology and Glycolytic Shift During Proximal Tubule Atrophy After Ischemic AKI. J. Am. Soc. Nephrol. 27 (11), 3356–3367. 10.1681/asn.2015020177 27000065 PMC5084876

[B72] Lawrence T. (2009). The Nuclear Factor NF-B Pathway in Inflammation. Cold Spring Harb. Perspect. Biol. 1 (6), a001651. 10.1101/cshperspect.a001651 20457564 PMC2882124

[B73] Li C.-M. Li M. Ye Z.-C. Huang J.-Y. Li Y. Yao Z.-Y. (2019). Circular RNA Expression Profiles in Cisplatin-Induced Acute Kidney Injury in Mice. Epigenomics 11 (10), 1191–1207. 10.2217/epi-2018-0167 31339054

[B74] Li J. Zhang Z. Wang L. Jiang L. Qin Z. Zhao Y. (2021a). Maresin 1 Attenuates Lipopolysaccharide-Induced Acute Kidney Injury via Inhibiting NOX4/ROS/NF-κB Pathway. Front. Pharmacol. 12. 10.3389/fphar.2021.782660 PMC8703041 34955852

[B75] Li T. Yu C. Zhuang S. (2021b). Histone Methyltransferase EZH2: A Potential Therapeutic Target for Kidney Diseases. Front. Physiol. 12, 640700. 10.3389/fphys.2021.640700 33679454 PMC7930071

[B76] Lin F. Xu L. Yuan R. Han S. Xie J. Jiang K. (2022a). Identification of Inflammatory Response and Alternative Splicing in Acute Kidney Injury and Experimental Verification of the Involvement of RNA-Binding Protein RBFOX1 in This Disease. Int. J. Mol. Med. 49 (3). 10.3892/ijmm.2022.5087 PMC8788925 35059728

[B77] Lin F. Y. Han S. T. Yu W. M. Rao T. Ruan Y. Yuan R. (2022b). microRNA‐486‐5p Is Implicated in the Cisplatin‐Induced Apoptosis and Acute Inflammation Response of Renal Tubular Epithelial Cells by Targeting HAT1. J Biochem. Mol. Tox, e23039. 10.1002/jbt.23039 35279909

[B78] Linkermann A. Chen G. Dong G. Kunzendorf U. Krautwald S. Dong Z. (2014). Regulated Cell Death in AKI. J.. Am. Soc. Nephrol. 25 (12), 2689–2701. 10.1681/asn.2014030262 24925726 PMC4243360

[B79] Liu H. Wu X. Luo J. Wang X. Guo H. Feng D. (2019a). Pterostilbene Attenuates Astrocytic Inflammation and Neuronal Oxidative Injury After Ischemia-Reperfusion by Inhibiting NF-Κb Phosphorylation. Front. Immunol. 10, 2408. 10.3389/fimmu.2019.02408 31681297 PMC6811521

[B80] Liu J. Jia Z. Gong W. (2021). Circulating Mitochondrial DNA Stimulates Innate Immune Signaling Pathways to Mediate Acute Kidney Injury. Front. Immunol. 12, 680648. 10.3389/fimmu.2021.680648 34248963 PMC8264283

[B81] Liu K. D. Goldstein S. L. Vijayan A. Parikh C. R. Kashani K. Okusa M. D. (2020a). AKI!Now Initiative: Recommendations for Awareness, Recognition, and Management of AKI. Clin. J. Am. Soc. Nephrol. 15 (12), 1838–1847. 10.2215/cjn.15611219 32317329 PMC7769012

[B82] Liu Z. Tang C. He L. Yang D. Cai J. Zhu J. (2020b). The Negative Feedback Loop of NF-κB/miR-376b/NFKBIZ in Septic Acute Kidney Injury. JCI Insight 5 (24). 10.1172/jci.insight.142272 PMC7819752 33328388

[B83] Liu Z. Wang Y. Shu S. Cai J. Tang C. Dong Z. (2019b). Non-Coding RNAs in Kidney Injury and Repair. Am. J. Physiology-Cell Physiology 317 (2), C177–c188. 10.1152/ajpcell.00048.2019 30969781

[B84] Lodde V. Murgia G. Simula E. R. Steri M. Floris M. Idda M. L. (2020). Long Noncoding RNAs and Circular RNAs in Autoimmune Diseases. Biomolecules 10 (7), 1044. 10.3390/biom10071044 PMC7407480 32674342

[B85] Lu H. Chen Y. Wang X. Yang Y. Ding M. Qiu F. (2022). Circular RNA HIPK3 Aggravates Sepsis-Induced Acute Kidney Injury via Modulating the microRNA-338/forkhead Box A1 Axis. Bioengineered 13 (3), 4798–4809. 10.1080/21655979.2022.2032974 35148669 PMC8974176

[B86] Lu S. Dong L. Jing X. Gen-Yang C. Zhan-Zheng Z. (2020). Abnormal lncRNA CCAT1/microRNA-155/SIRT1 axis Promoted Inflammatory Response and Apoptosis of Tubular Epithelial Cells in LPS Caused Acute Kidney Injury. Mitochondrion 53, 76–90. 10.1016/j.mito.2020.03.010 32243922

[B87] Lu T. X. Rothenberg M. E. (2018). MicroRNA. J. Allergy Clin. Immunol. 141 (4), 1202–1207. 10.1016/j.jaci.2017.08.034 29074454 PMC5889965

[B88] Lv L.-L. Feng Y. Wu M. Wang B. Li Z.-L. Zhong X. (2020). Exosomal miRNA-19b-3p of Tubular Epithelial Cells Promotes M1 Macrophage Activation in Kidney Injury. Cell Death Differ. 27 (1), 210–226. 10.1038/s41418-019-0349-y 31097789 PMC7206053

[B89] Ma T. Jia H. Ji P. He Y. Chen L. (2021a). Identification of the Candidate lncRNA Biomarkers for Acute Kidney Injury: A Systematic Review and Meta-Analysis. Expert Rev. Mol. Diagnostics 21 (1), 77–89. 10.1080/14737159.2021.1873131 33612038

[B90] Ma X. Zhu G. Jiao T. Shao F. (2021b). Effects of Circular RNA Ttc3/miR-148a/Rcan2 Axis on Inflammation and Oxidative Stress in Rats with Acute Kidney Injury Induced by Sepsis. Life Sci. 272, 119233. 10.1016/j.lfs.2021.119233 33600863

[B91] Markó L. Vigolo E. Hinze C. Park J.-K. Roël G. Balogh A. (2016). Tubular Epithelial NF-Κb Activity Regulates Ischemic AKI. J. Am. Soc. Nephrol. 27 (9), 2658–2669. 10.1681/asn.2015070748 26823548 PMC5004652

[B92] Matsui M. Corey D. R. (2017). Non-Coding RNAs as Drug Targets. Nat. Rev. Drug Discov. 16 (3), 167–179. 10.1038/nrd.2016.117 27444227 PMC5831170

[B93] McSweeney K. R. Gadanec L. K. Qaradakhi T. Ali B. A. Zulli A. Apostolopoulos V. (2021). Mechanisms of Cisplatin-Induced Acute Kidney Injury: Pathological Mechanisms, Pharmacological Interventions, and Genetic Mitigations. Cancers 13 (7), 1572. 10.3390/cancers13071572 33805488 PMC8036620

[B94] McWilliam S. J. Wright R. D. Welsh G. I. Tuffin J. Budge K. L. Swan L. (2021). The Complex Interplay Between Kidney Injury and Inflammation. Clin. Kidney J. 14 (3), 780–788. 10.1093/ckj/sfaa164 33777361 PMC7986351

[B95] Mehta R. L. Kellum J. A. Shah S. V. Molitoris B. A. Ronco C. Warnock D. G. (2007). Acute Kidney Injury Network: Report of an Initiative to Improve Outcomes in Acute Kidney Injury. Crit. Care 11 (2), R31. 10.1186/cc5713 17331245 PMC2206446

[B96] Miao S. Lv C. Liu Y. Zhao J. Li T. Wang C. (2020). Pharmacologic Blockade of 15-PGDH Protects Against Acute Renal Injury Induced by LPS in Mice. Front. Physiol. 11, 138. 10.3389/fphys.2020.00138 32231583 PMC7082810

[B97] Nechemia-Arbely Y. Barkan D. Pizov G. Shriki A. Rose-John S. Galun E. (2008). IL-6/IL-6R Axis Plays a Critical Role in Acute Kidney Injury. J. Am. Soc. Nephrol. 19 (6), 1106–1115. 10.1681/asn.2007070744 18337485 PMC2396933

[B98] O'Neill L. A. J. Golenbock D. Bowie A. G. (2013). The History of Toll-like Receptors - Redefining Innate Immunity. Nat. Rev. Immunol. 13 (6), 453–460. 10.1038/nri3446 23681101

[B99] Oeckinghaus A. Hayden M. S. Ghosh S. (2011). Crosstalk in NF-Κb Signaling Pathways. Nat. Immunol. 12 (8), 695–708. 10.1038/ni.2065 21772278

[B100] Ostermann M. Bellomo R. Burdmann E. A. Doi K. Endre Z. H. Goldstein S. L. (2020). Controversies in Acute Kidney Injury: Conclusions from a Kidney Disease: Improving Global Outcomes (KDIGO) Conference. Kidney Int. 98 (2), 294–309. 10.1016/j.kint.2020.04.020 32709292 PMC8481001

[B101] Ozkok A. Edelstein C. L. (2014). Pathophysiology of Cisplatin-Induced Acute Kidney Injury. BioMed Res. Int. 2014, 1–17. 10.1155/2014/967826 PMC4140112 25165721

[B102] Patop I. L. Wüst S. Kadener S. (2019). Past, Present, and Future of Circ RNA S. Embo J. 38 (16), e100836. 10.15252/embj.2018100836 31343080 PMC6694216

[B103] Peerapornratana S. Manrique-Caballero C. L. Gómez H. Kellum J. A. (2019). Acute Kidney Injury from Sepsis: Current Concepts, Epidemiology, Pathophysiology, Prevention and Treatment. Kidney Int. 96 (5), 1083–1099. 10.1016/j.kint.2019.05.026 31443997 PMC6920048

[B104] Pegtel D. M. Gould S. J. (2019). Exosomes. Annu. Rev. Biochem. 88, 487–514. 10.1146/annurev-biochem-013118-111902 31220978

[B105] Perazella M. A. (2019). Drug-induced Acute Kidney Injury. Curr. Opin. Crit. Care 25 (6), 550–557. 10.1097/mcc.0000000000000653 31483318

[B106] Petejova N. Martinek A. Zadrazil J. Kanova M. Klementa V. Sigutova R. (2020). Acute Kidney Injury in Septic Patients Treated by Selected Nephrotoxic Antibiotic Agents-Pathophysiology and Biomarkers-A Review. Ijms 21 (19), 7115. 10.3390/ijms21197115 PMC7583998 32993185

[B107] Pidwill G. R. Gibson J. F. Cole J. Renshaw S. A. Foster S. J. (2020). The Role of Macrophages in *Staphylococcus aureus* Infection. Front. Immunol. 11, 620339. 10.3389/fimmu.2020.620339 33542723 PMC7850989

[B108] Pinheiro K. H. E. Azêdo F. A. Areco K. C. N. Laranja S. M. R. (2019). Risk Factors and Mortality in Patients with Sepsis, Septic and Non Septic Acute Kidney Injury in ICU. Braz. J. Nephrol. 41 (4), 462–471. 10.1590/2175-8239-jbn-2018-0240 PMC6979581 31528980

[B109] Poston J. T. Koyner J. L. (2019). Sepsis Associated Acute Kidney Injury. Bmj 364, k4891. 10.1136/bmj.k4891 30626586 PMC6890472

[B110] Prasada R. Muktesh G. Samanta J. Sarma P. Singh S. Arora S. K. (2020). Natural History and Profile of Selective Cytokines in Patients of Acute Pancreatitis with Acute Kidney Injury. Cytokine 133, 155177. 10.1016/j.cyto.2020.155177 32593952

[B111] Qian X. Zhao J. Yeung P. Y. Zhang Q. C. Kwok C. K. (2019). Revealing lncRNA Structures and Interactions by Sequencing-Based Approaches. Trends Biochem. Sci. 44 (1), 33–52. 10.1016/j.tibs.2018.09.012 30459069

[B112] Rabb H. Griffin M. D. McKay D. B. Swaminathan S. Pickkers P. Rosner M. H. (2016). Inflammation in AKI: Current Understanding, Key Questions, and Knowledge Gaps. J. Am. Soc. Nephrol. 27 (2), 371–379. 10.1681/asn.2015030261 26561643 PMC4731128

[B113] Ren G. L. Zhu J. Li J. Meng X. M. (2019). Noncoding RNAs in Acute Kidney Injury. J. Cell. Physiology 234 (3), 2266–2276. 10.1002/jcp.27203 30146769

[B114] Rosales C. (2018). Neutrophil: A Cell with Many Roles in Inflammation or Several Cell Types? Front. Physiol. 9, 113. 10.3389/fphys.2018.00113 29515456 PMC5826082

[B115] Rossaint J. Zarbock A. (2016). Acute Kidney Injury: Definition, Diagnosis and Epidemiology. Minerva Urol. Nefrol. 68 (1), 49–57. 26364570

[B116] Saliminejad K. Khorram Khorshid H. R. Soleymani Fard S. Ghaffari S. H. (2019). An Overview of microRNAs: Biology, Functions, Therapeutics, and Analysis Methods. J. Cell. Physiology 234 (5), 5451–5465. 10.1002/jcp.27486 30471116

[B117] Sato Y. Takahashi M. Yanagita M. (2020). Pathophysiology of AKI to CKD Progression. Seminars Nephrol. 40 (2), 206–215. 10.1016/j.semnephrol.2020.01.011 32303283

[B118] Sato Y. Yanagita M. (2018). Immune Cells and Inflammation in AKI to CKD Progression. Am. J. Physiology-Renal Physiology 315 (6), F1501–f1512. 10.1152/ajprenal.00195.2018 30156114

[B119] Schmitz S. U. Grote P. Herrmann B. G. (2016). Mechanisms of Long Noncoding RNA Function in Development and Disease. Cell. Mol. Life Sci. 73 (13), 2491–2509. 10.1007/s00018-016-2174-5 27007508 PMC4894931

[B120] See E. J. See K. Glassford N. Bailey M. Johnson D. W. Polkinghorne K. R. (2019). Long-Term Risk of Adverse Outcomes after Acute Kidney Injury: a Systematic Review and Meta-Analysis of Cohort Studies Using Consensus Definitions of Exposure. Kidney Int. 95 (1), 160–172. 10.1016/j.kint.2018.08.036 30473140

[B121] Shi C. Zhao Y. Li Q. Li J. (2021). lncRNA SNHG14 Plays a Role in Sepsis-Induced Acute Kidney Injury by Regulating miR-93. Mediat. Inflamm. 2021, 1–10. 10.1155/2021/5318369 PMC7806393 33505213

[B122] Shi Y. Sun C. F. Ge W. H. Du Y. P. Hu N. B. (2020). Circular RNA VMA21 Ameliorates Sepsis‐Associated Acute Kidney Injury by Regulating miR‐9‐3p/SMG1/Inflammation Axis and Oxidative Stress. J. Cell. Mol. Med. 24 (19), 11397–11408. 10.1111/jcmm.15741 32827242 PMC7576305

[B123] Simion V. Zhou H. Pierce J. B. Yang D. Haemmig S. Tesmenitsky Y. (2020). LncRNA VINAS Regulates Atherosclerosis by Modulating NF-Κb and MAPK Signaling. JCI Insight 5 (21). 10.1172/jci.insight.140627 PMC7710319 33021969

[B124] Singbartl K. Kellum J. A. (2012). AKI in the ICU: Definition, Epidemiology, Risk Stratification, and Outcomes. Kidney Int. 81 (9), 819–825. 10.1038/ki.2011.339 21975865

[B125] Song A. Zhang C. Meng X. (2021). Mechanism and Application of Metformin in Kidney Diseases: An Update. Biomed. Pharmacother. 138, 111454. 10.1016/j.biopha.2021.111454 33714781

[B126] Song N. Zhang T. Xu X. Lu Z. Yu X. Fang Y. (2018). miR-21 Protects against Ischemia/Reperfusion-Induced Acute Kidney Injury by Preventing Epithelial Cell Apoptosis and Inhibiting Dendritic Cell Maturation. Front. Physiol. 9, 790. 10.3389/fphys.2018.00790 30013485 PMC6036242

[B127] Sun B. Q. Sui Y. D. Huang H. Zou X. B. Chen S. C. Yu Z. K. (2019). Effect of lncRNA CRNDE on Sepsis-Related Kidney Injury Through the TLR3/NF-Κb Pathway. Eur. Rev. Med. Pharmacol. Sci. 23 (23), 10489–10497. 10.26355/eurrev_201912_19688 31841203

[B128] Tan J. Fan J. He J. Zhao L. Tang H. (2020). Knockdown of LncRNA DLX6-AS1 Inhibits HK-2 Cell Pyroptosis via Regulating miR-223-3p/NLRP3 Pathway in Lipopolysaccharide-Induced Acute Kidney Injury. J. Bioenerg. Biomembr. 52 (5), 367–376. 10.1007/s10863-020-09845-5 32666494

[B129] Tang C. Han H. Liu Z. Liu Y. Yin L. Cai J. (2019). Activation of BNIP3-Mediated Mitophagy Protects Against Renal Ischemia-Reperfusion Injury. Cell Death Dis. 10 (9), 677. 10.1038/s41419-019-1899-0 31515472 PMC6742651

[B130] Thomas M. E. Blaine C. Dawnay A. Devonald M. A. J. Ftouh S. Laing C. (2015). The Definition of Acute Kidney Injury and its Use in Practice. Kidney Int. 87 (1), 62–73. 10.1038/ki.2014.328 25317932

[B131] Van Opdenbosch N. Lamkanfi M. (2019). Caspases in Cell Death, Inflammation, and Disease. Immunity 50 (6), 1352–1364. 10.1016/j.immuni.2019.05.020 31216460 PMC6611727

[B132] Venkatachalam M. A. Weinberg J. M. Kriz W. Bidani A. K. (2015). Failed Tubule Recovery, AKI-CKD Transition, and Kidney Disease Progression. J. Am. Soc. Nephrol. 26 (8), 1765–1776. 10.1681/asn.2015010006 25810494 PMC4520181

[B133] Verstrepen L. Bekaert T. Chau T.-L. Tavernier J. Chariot A. Beyaert R. (2008). TLR-4, IL-1R and TNF-R Signaling to NF-Κb: Variations on a Common Theme. Cell. Mol. Life Sci. 65 (19), 2964–2978. 10.1007/s00018-008-8064-8 18535784 PMC11131687

[B134] Volarevic V. Djokovic B. Jankovic M. G. Harrell C. R. Fellabaum C. Djonov V. (2019). Molecular Mechanisms of Cisplatin-Induced Nephrotoxicity: A Balance on the Knife Edge Between Renoprotection and Tumor Toxicity. J. Biomed. Sci. 26 (1), 25. 10.1186/s12929-019-0518-9 30866950 PMC6417243

[B135] Wang F. Zhang F. Tian Q. Sheng K. (2022). CircVMA21 Ameliorates Lipopolysaccharide (LPS)-Induced HK-2 Cell Injury Depending on the Regulation of miR-7-5p/PPARA. Autoimmunity 55 (2), 136–146. 10.1080/08916934.2021.2012764 34894921

[B136] Wang H. Mou H. Xu X. Liu C. Zhou G. Gao B. (2021a). LncRNA KCNQ1OT1 (Potassium Voltage-Gated Channel Subfamily Q Member 1 Opposite Strand/Antisense Transcript 1) Aggravates Acute Kidney Injury by Activating p38/NF-Κb Pathway via miR-212-3p/MAPK1 (Mitogen-Activated Protein Kinase 1) Axis in Sepsis. Bioengineered 12 (2), 11353–11368. 10.1080/21655979.2021.2005987 34783627 PMC8810185

[B137] Wang J. Jiao P. Wei X. Zhou Y. (2021b). Silencing Long Non-Coding RNA Kcnq1ot1 Limits Acute Kidney Injury by Promoting miR-204-5p and Blocking the Activation of NLRP3 Inflammasome. Front. Physiol. 12, 721524. 10.3389/fphys.2021.721524 34858199 PMC8632456

[B138] Wang J. Song J. Li Y. Shao J. Xie Z. Sun K. (2020a). Down-Regulation of LncRNA CRNDE Aggravates Kidney Injury via Increasing MiR-181a-5p in Sepsis. Int. Immunopharmacol. 79, 105933. 10.1016/j.intimp.2019.105933 31877497

[B139] Wang L. Cao Q. M. (2022). Long Non‐coding RNA XIST Alleviates Sepsis‐Induced Acute Kidney Injury Through Inhibiting Inflammation and Cell Apoptosis via Regulating miR‐155‐5p/WWC1 axis. Kaohsiung J Med Scie 38 (1), 6–17. 10.1002/kjm2.12442 PMC11896158 34431595

[B140] Wang M. Wei J. Shang F. Zang K. Zhang P. (2021c). Down-Regulation of lncRNA SNHG5 Relieves Sepsis-Induced Acute Kidney Injury by Regulating the miR-374a-3p/TLR4/NF-Κb Pathway. J. Biochem. 169 (5), 575–583. 10.1093/jb/mvab008 33479745

[B141] Wang X. Li Y. Zhao Z. Meng Y. Bian J. Bao R. (2019). IGFBP7 Regulates Sepsis-Induced Epithelial-Mesenchymal Transition Through ERK1/2 Signaling. Acta Biochim. Biophys. Sin. (Shanghai) 51 (8), 799–806. 10.1093/abbs/gmz072 31287495

[B142] Wang Y. Liu Z. Shu S. Cai J. Tang C. Dong Z. (2020b). AMPK/mTOR Signaling in Autophagy Regulation During Cisplatin-Induced Acute Kidney Injury. Front. Physiol. 11, 619730. 10.3389/fphys.2020.619730 33391038 PMC7773913

[B143] Wang Y. Zhang H. Chen Q. Jiao F. Shi C. Pei M. (2020c). TNF‐α/HMGB1 Inflammation Signalling Pathway Regulates Pyroptosis During Liver Failure and Acute Kidney Injury. Cell Prolif. 53 (6), e12829. 10.1111/cpr.12829 32419317 PMC7309595

[B144] Wilflingseder J. Willi M. Lee H. K. Olauson H. Jankowski J. Ichimura T. (2020). Enhancer and Super-Enhancer Dynamics in Repair After Ischemic Acute Kidney Injury. Nat. Commun. 11 (1), 3383. 10.1038/s41467-020-17205-5 32636391 PMC7341735

[B145] Winkle M. El-Daly S. M. Fabbri M. Calin G. A. (2021). Noncoding RNA Therapeutics - Challenges and Potential Solutions. Nat. Rev. Drug Discov. 20 (8), 629–651. 10.1038/s41573-021-00219-z 34145432 PMC8212082

[B146] Wu H. Wang J. Ma Z. (2020). Long Noncoding RNA HOXA‐AS2 Mediates microRNA‐106b‐5p to Repress Sepsis‐Engendered Acute Kidney Injury. J. Biochem. Mol. Toxicol. 34 (4), e22453. 10.1002/jbt.22453 32048402

[B147] Xiao C. Zhao H. Zhu H. Zhang Y. Su Q. Zhao F. (2020). Tisp40 Induces Tubular Epithelial Cell GSDMD-Mediated Pyroptosis in Renal Ischemia-Reperfusion Injury via NF-Κb Signaling. Front. Physiol. 11, 906. 10.3389/fphys.2020.00906 32903383 PMC7438479

[B148] Xie Z. Wei L. Chen J. Chen Z. (2022). LncRNA NORAD Deficiency Alleviates Kidney Injury in Mice and Decreases the Inflammatory Response and Apoptosis of Lipopolysaccharide-Stimulated HK-2 Cells via the miR-577/GOLPH3 axis. Cytokine 153, 155844. 10.1016/j.cyto.2022.155844 35255377

[B149] Xu H.-P. Ma X.-Y. Yang C. (2021a). Circular RNA TLK1 Promotes Sepsis-Associated Acute Kidney Injury by Regulating Inflammation and Oxidative Stress through miR-106a-5p/HMGB1 Axis. Front. Mol. Biosci. 8, 660269. 10.3389/fmolb.2021.660269 34250012 PMC8266998

[B150] Xu L. Cao H. Xu P. Nie M. Zhao C. (2022). Circ_0114427 Promotes LPS-Induced Septic Acute Kidney Injury by Modulating miR-495-3p/TRAF6 through the NF-Κb Pathway. Autoimmunity 55 (1), 52–64. 10.1080/08916934.2021.1995861 34730059

[B151] Xu Y. Li X. Li H. Zhong L. Lin Y. Xie J. (2021b). Circ_0023404 Sponges miR‐136 to Induce HK‐2 Cells Injury Triggered by Hypoxia/Reoxygenation via Up‐Regulating IL‐6R. J. Cell Mol. Med. 25 (11), 4912–4921. 10.1111/jcmm.15986 33942982 PMC8178261

[B152] Xu Y. Ma H. Shao J. Wu J. Zhou L. Zhang Z. (2015). A Role for Tubular Necroptosis in Cisplatin-Induced AKI. J. Am. Soc. Nephrol. 26 (11), 2647–2658. 10.1681/asn.2014080741 25788533 PMC4625668

[B153] Xue Q. Yang L. Wang H. Han S. (2021). Silence of Long Noncoding RNA SNHG14 Alleviates Ischemia/Reperfusion-Induced Acute Kidney Injury by Regulating miR-124-3p/MMP2 Axis. BioMed Res. Int. 2021, 1–13. 10.1155/2021/8884438 33490282 PMC7803415

[B154] Yang C. Yang C. Huang Z. Zhang J. Chen N. Guo Y. (2022a). Reduced Expression of MiR-125a-5p Aggravates LPS-Induced Experimental Acute Kidney Injury Pathology by Targeting TRAF6. Life Sci. 288, 119657. 10.1016/j.lfs.2021.119657 34048808

[B155] Yang J. Wu L. Liu S. Hu X. Wang Q. Fang L. (2021a). Long Non-coding RNA NEAT1 Promotes Lipopolysaccharide-Induced Injury in Human Tubule Epithelial Cells by Regulating miR-93-5p/TXNIP Axis. Med. Microbiol. Immunol. 210 (2-3), 121–132. 10.1007/s00430-021-00705-6 33885954

[B156] Yang M. Lu H. Liu J. Wu S. Kim P. Zhou X. (2022b). lncRNAfunc: A Knowledgebase of lncRNA Function in Human Cancer. Nucleic Acids Res. 50 (D1), D1295–d1306. 10.1093/nar/gkab1035 34791419 PMC8728133

[B157] Yang N. Wang H. Zhang L. Lv J. Niu Z. Liu J. (2021b). Long Non-Coding RNA SNHG14 Aggravates LPS-Induced Acute Kidney Injury Through Regulating miR-495-3p/HIPK1. Acta Biochim. Biophys. Sin. (Shanghai) 53 (6), 719–728. 10.1093/abbs/gmab034 33856026

[B158] Yeung F. Hoberg J. E. Ramsey C. S. Keller M. D. Jones D. R. Frye R. A. (2004). Modulation of NF-κb-Dependent Transcription and Cell Survival by the SIRT1 Deacetylase. Embo J. 23 (12), 2369–2380. 10.1038/sj.emboj.7600244 15152190 PMC423286

[B159] Yu J. Xie D. Huang N. Zhou Q. (2021). Circular RNAs as Novel Diagnostic Biomarkers and Therapeutic Targets in Kidney Disease. Front. Med. 8, 714958. 10.3389/fmed.2021.714958 PMC8481637 34604256

[B160] Zang J. Lu D. Xu A. (2020). The Interaction of circRNAs and RNA Binding Proteins: An Important Part of circRNA Maintenance and Function. J. Neurosci. Res. 98 (1), 87–97. 10.1002/jnr.24356 30575990

[B161] Zhang B. Zeng M. Li M. Kan Y. Li B. Xu R. (2019). Protopine Protects Mice against LPS-Induced Acute Kidney Injury by Inhibiting Apoptosis and Inflammation via the TLR4 Signaling Pathway. Molecules 25 (1), 15. 10.3390/molecules25010015 PMC6982873 31861525

[B162] Zhang D. Qi B. Li D. Feng J. Huang X. Ma X. (2020a). Phillyrin Relieves Lipopolysaccharide-Induced AKI by Protecting against Glycocalyx Damage and Inhibiting Inflammatory Responses. Inflammation 43 (2), 540–551. 10.1007/s10753-019-01136-5 31832909 PMC7095384

[B163] Zhang L. He S. Wang Y. Zhu X. Shao W. Xu Q. (2020b). miRNA-20a Suppressed Lipopolysaccharide‐Induced HK‐2 Cells Injury via NFκB and ERK1/2 Signaling by Targeting CXCL12. Mol. Immunol. 118, 117–123. 10.1016/j.molimm.2019.12.009 31874343

[B164] Zhang Q. Wang L. Wu M. Liu X. Zhu Y. Zhu J. (2021). Humanized Anti-TLR4 M-onoclonal A-ntibody A-meliorates L-ipopolysaccharide-Related A-cute K-idney I-njury by I-nhibiting TLR4/NF-κB S-ignaling. Mol. Med. Rep. 24 (2). 10.3892/mmr.2021.12245 PMC8240183 34184086

[B165] Zhang R. Zhu Y. Li Y. Liu W. Yin L. Yin S. (2020c). Human Umbilical Cord Mesenchymal Stem Cell Exosomes Alleviate Sepsis-Associated Acute Kidney Injury via Regulating microRNA-146b Expression. Biotechnol. Lett. 42 (4), 669–679. 10.1007/s10529-020-02831-2 32048128

[B166] Zhang Y. Li C. Guan C. Zhou B. Wang L. Yang C. (2020d). MiR-181d-5p Targets KLF6 to Improve Ischemia/Reperfusion-Induced AKI Through Effects on Renal Function, Apoptosis, and Inflammation. Front. Physiol. 11, 510. 10.3389/fphys.2020.00510 32581828 PMC7295155

[B167] Zhao H. Wu L. Yan G. Chen Y. Zhou M. Wu Y. (2021a). Inflammation and Tumor Progression: Signaling Pathways and Targeted Intervention. Sig Transduct. Target Ther. 6 (1), 263. 10.1038/s41392-021-00658-5 PMC8273155 34248142

[B168] Zhao M. Wang Y. Li L. Liu S. Wang C. Yuan Y. (2021b). Mitochondrial ROS Promote Mitochondrial Dysfunction and Inflammation in Ischemic Acute Kidney Injury by Disrupting TFAM-Mediated mtDNA Maintenance. Theranostics 11 (4), 1845–1863. 10.7150/thno.50905 33408785 PMC7778599

[B169] Zhao W. Zhang L. Chen R. Lu H. Sui M. Zhu Y. (2018). SIRT3 Protects Against Acute Kidney Injury via AMPK/mTOR-Regulated Autophagy. Front. Physiol. 9, 1526. 10.3389/fphys.2018.01526 30487750 PMC6246697

[B170] Zheng C. Wu D. Shi S. Wang L. (2021). miR-34b-5p Promotes Renal Cell Inflammation and Apoptosis by Inhibiting Aquaporin-2 in Sepsis-Induced Acute Kidney Injury. Ren. Fail. 43 (1), 291–301. 10.1080/0886022x.2021.1871922 33494641 PMC7850462

[B171] Zhou X. Jiang K. Luo H. Wu C. Yu W. Cheng F. (2020). Novel lncRNA XLOC_032768 Alleviates Cisplatin-Induced Apoptosis and Inflammatory Response of Renal Tubular Epithelial Cells Through TNF-α. Int. Immunopharmacol. 83, 106472. 10.1016/j.intimp.2020.106472 32278129

[B172] Zhou Y. Qing M. Xu M. (2021a). Circ-BNIP3L Knockdown Alleviates LPS-Induced Renal Tubular Epithelial Cell Injury During Sepsis-Associated Acute Kidney Injury by miR-370-3p/MYD88 axis. J. Bioenerg. Biomembr. 53 (6), 665–677. 10.1007/s10863-021-09925-0 34731384

[B173] Zhou Y. Sun W. Qin Z. Guo S. Kang Y. Zeng S. (2021b). LncRNA Regulation: New Frontiers in Epigenetic Solutions to Drug Chemoresistance. Biochem. Pharmacol. 189, 114228. 10.1016/j.bcp.2020.114228 32976832

[B174] Zhu H. Ren A. Zhou K. Chen Q. Zhang M. Liu J. (2020). Impact of Dexmedetomidine Infusion on Postoperative Acute Kidney Injury in Elderly Patients Undergoing Major Joint Replacement: A Retrospective Cohort Study. Dddt 14, 4695–4701. 10.2147/dddt.S278342 33173279 PMC7646437

[B175] Zhu J. Fu H. Wu Y. Zheng X. (2013). Function of lncRNAs and Approaches to lncRNA-Protein Interactions. Sci. China Life Sci. 56 (10), 876–885. 10.1007/s11427-013-4553-6 24091684

